# Inflammatory and cholesterol risks and rates of major cardiovascular events among patients with atherosclerotic cardiovascular disease in routine care

**DOI:** 10.1093/ehjopen/oeag023

**Published:** 2026-02-17

**Authors:** Faizan Mazhar, Davide Capodanno, Paul Hjemdahl, Arvid Sjölander, Sofia Gerward, Jimmi Mathisen, Oscar Plunde, Vijay Kunadian, Tomas Jernberg, Juan-Jesus Carrero

**Affiliations:** Department of Medical Epidemiology and Biostatistics, Campus Solna, Karolinska Institutet, Nobels väg 12A, 17165 Solna, Stockholm, Sweden; Division of Cardiology, Azienda Ospedaliero-Universitaria Policlinico ‘G. Rodolico-San Marco’, University of Catania, Via S.Sofia, 78 - 95123 Catania, Italy; Department of Medicine Solna, Clinical Epidemiology/Clinical Pharmacology, Karolinska Institutet and Karolinska University Hospital, Framstegsgatan, 171 64 Solna, Stockholm, Sweden; Department of Medical Epidemiology and Biostatistics, Campus Solna, Karolinska Institutet, Nobels väg 12A, 17165 Solna, Stockholm, Sweden; Novo Nordisk A/S, Vandtårnsvej 108, 2860 Søborg, Denmark; Novo Nordisk A/S, Vandtårnsvej 108, 2860 Søborg, Denmark; Novo Nordisk A/S, Vandtårnsvej 108, 2860 Søborg, Denmark; Translational and Clinical Research Institute, Faculty of Medical Sciences, Newcastle University and Cardiothoracic Centre, Freeman Hospital, Newcastle upon Tyne Hospitals NHS Foundation Trust, Newcastle upon Tyne NE1 7RU, UK; Department of Clinical Sciences, Danderyd Hospital, Karolinska Institutet, Entrévägen 2, 182 88 Danderyd, Stockholm, Sweden; Department of Medical Epidemiology and Biostatistics, Campus Solna, Karolinska Institutet, Nobels väg 12A, 17165 Solna, Stockholm, Sweden; Division of Nephrology, Department of Clinical Sciences, Danderyd Hospital, Entrévägen 2, 182 88 Danderyd, Sweden

**Keywords:** SCREAM, Residual risk, Statins, Myocardial infarction, Heart failure, Stroke

## Abstract

**Aims:**

Inflammation and hyperlipidaemia play a pivotal role in atherosclerotic cardiovascular disease (ASCVD), and inflammatory risk may outweigh cholesterol risk among statin-treated patients. However, it is unclear how these risks relate to ASCVD outcomes in a real-world population.

**Methods and results:**

Observational study of 39 638 ASCVD adults in Stockholm’s healthcare (2007–21) who underwent routine testing for high-sensitivity C-reactive protein (hsCRP) and low-density lipoprotein cholesterol (LDL-C). Groups were defined by LDL-C (≥1.8 vs. < 1.8 mmol/L) and hsCRP (≥2 vs. < 2 mg/L): as low risk, high cholesterol risk (CR) alone, high inflammatory risk (IR) alone, and combined high cholesterol and inflammatory risk (CIR). Primary outcome was major adverse cardiovascular (CV) events (MACE); secondary outcomes included all-cause death, CV death, and heart failure (HF) hospitalization. Mean age at baseline was 69 years, 61% were men, 19.4% had chronic kidney disease (CKD), and 61% were receiving lipid-lowering therapy (LLT). Over follow-up (median 4.5 years), 5349 MACE, 7955 deaths (2088 CV deaths) and 4286 HF hospitalizations occurred. Compared with patients with low risk, those with IR or CIR experienced the highest MACE risk (HR 1.39; 95% CI 1.26–1.54 for CIR, HR 1.18; 1.05–1.33 for IR), followed by CR (HR 1.12; 1.01–1.24). Elevated hsCRP, with or without elevated LDL-C, was strongly associated with secondary outcomes, while CR alone was not. Patterns were generally consistent across CKD and LLT subgroups.

**Conclusion:**

In routine care high inflammatory risk, alone or with high cholesterol risk, is a stronger predictor of adverse outcomes than high cholesterol alone.

## Graphical Abstract

**Graphical Abstract:**
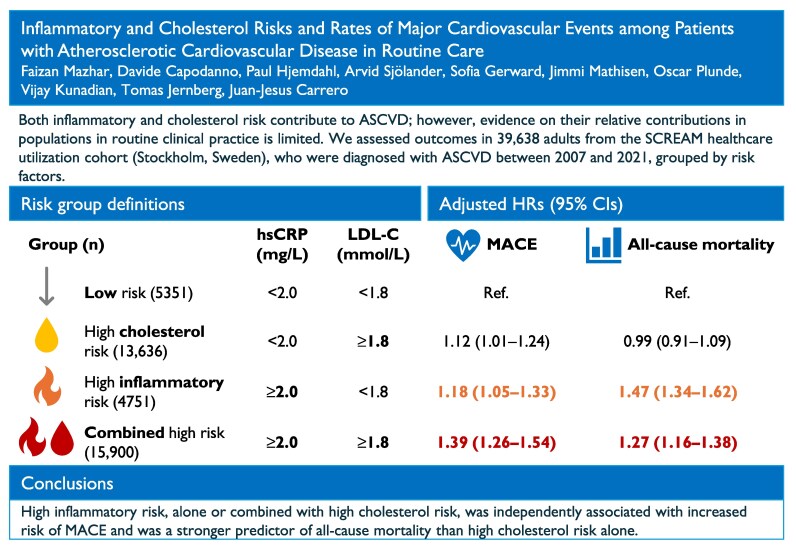

## Introduction

Atherosclerotic cardiovascular disease (ASCVD) remains the leading cause of morbidity and mortality worldwide.^1^ The causal role of low-density lipoprotein cholesterol (LDL-C) in atherogenesis is well established,^2^ and statins are the cornerstone of lipid-lowering therapy (LLT). However, a substantial residual risk remains even in patients who achieve optimal LDL-C levels, indicating the need for additional strategies beyond cholesterol lowering.^3^

Mounting evidence underscores the importance of inflammation as an independent predictor of ASCVD progression and adverse cardiovascular outcomes.^3^ Landmark clinical trials have demonstrated that targeting both lipid and inflammatory pathways yields incremental benefits,^4^ reinforcing the importance of inflammation in ASCVD pathogenesis. This has led to growing recognition of cholesterol risk and inflammatory risks as distinct yet interrelated targets for risk reduction in secondary prevention strategies.^5–9^

In previous work, we found that that up to 60% of patients with ASCVD in routine care exhibit high levels of systemic inflammation, as measured by high-sensitivity C-reactive protein (hsCRP) above 2 mg/L.^10^ Despite this common prevalence, inflammatory biomarkers are still not integrated in the standard monitoring protocols for secondary ASCVD prevention. This may be partly due to uncertainty about the comparative prognostic value of LDL-C and hsCRP in the diverse populations seen in everyday clinical practice. In fact, most available evidence stems from randomized controlled trials involving narrowly selected, statin-treated populations, limiting generalizability to real-world settings where treatment patterns and baseline risks vary substantially.

The prognostic usefulness of inflammatory risk and cholesterol risk in the absence of LLT is not well studied. Furthermore, this question may be especially relevant for high-risk subgroups such as individuals with chronic kidney disease (CKD), who often present with both dysregulated lipid profiles and elevated inflammatory burden. *Post hoc* analyses from the CANTOS trial suggested that in statin-treated patients with CKD, residual inflammatory risk—not residual cholesterol risk—may be the dominant predictor of cardiovascular outcomes.^11^ However, these findings require confirmation, as they were based on selective trial cohorts with a limited number of people with CKD as per exclusion criteria.

Evaluating the individual and joint prognostic value of LDL-C and hsCRP in patients with ASCVD receiving routine care may inform discussions on the value of hsCRP monitoring. To address these gaps, we leveraged comprehensive real-world data from the Swedish SCREAM (Stockholm CREAtinine Measurements) project, with particular focus on the largely unexplored subgroups of patients with CKD or those not receiving LLT.

## Methods

### Data source

This study used data from the SCREAM project, a comprehensive healthcare utilization cohort from the Stockholm region of Sweden.^12^ In this region, a single healthcare provider delivers universal, tax-funded care to approximately 20–25% of the Swedish population. SCREAM is linked to regional and national administrative registers containing complete information on demographics, healthcare utilization, laboratory tests, dispensed medications, diagnoses, and vital status, with no loss to follow-up. The study was approved by the regional ethical review board in Stockholm. Informed consent was not required, as data were de-identified by the Swedish Board of Health and Welfare.

### Study design and study population

We included patients aged ≥18 years with an incident diagnosis of ASCVD between 1 January 2007, and 31 December 2021 (the most recent data available in SCREAM). ASCVD was defined as a clinical diagnosis of coronary, cerebrovascular, or peripheral artery disease using ICD-10 codes (see Supplementary material online, *Table S1*). The date of the first qualifying diagnosis was defined as the cohort entry date (see Supplementary material online, *Figure S1*). All subsequent measurements of hsCRP and LDL-C were extracted.

Because laboratory testing in real-world practice is performed at physicians’ discretion and for diverse clinical indications, we applied several patient- and test-level exclusion criteria to reduce potential biases related to healthcare utilization (see Supplementary material online, *Supplemental methods* and Supplementary material online, *Table S2*). To define baseline exposure, pairs of hsCRP and LDL-C measurements, performed on the same day or within 30 days of each other, were identified. Then, the later test date in the earliest eligible pair was designated as the exposure start date. At this time point, we excluded patients with conditions or medications known to affect systemic inflammation (e.g. chronic infections, recent cancer diagnoses, corticosteroids, or immunosuppressive agents).

To capture baseline (‘background’) systemic inflammation and lipid status while reducing the influence of short-term biological variability, we defined hsCRP and LDL-C levels using all eligible tests obtained within a prespecified 3-month ascertainment (landmark) window following the exposure start date. For participants with multiple measurements within this window, we used the geometric mean, which is well suited to right-skewed biomarkers such as hsCRP and reduces the influence of occasional extreme values, providing a more robust estimate of central tendency. Because follow-up began at the end of the ascertainment window (the index date), we excluded patients who died within 3 months of the exposure start date, had fewer than 3 months of follow-up, or lacked creatinine measurements in the year prior (necessary to estimate kidney function). The end of this window was designated as the index date, on which baseline covariates were measured and follow-up began (see Supplementary material online, *Figure S1*).

### Study exposures

Primary exposures were hsCRP (as a proxy for systemic inflammation) and LDL-C (as a marker of lipid status). Laboratory services in the Stockholm region are centralized and subject to routine quality control by the government agency EQUALIS (www.equalis.se/en), ensuring standardization, reproducibility, and consistency across the region’s unified healthcare. hsCRP was measured in plasma samples using immunochemistry or turbidimetry, and all assays had detection limits ≤1 mg/L. The majority of LDL-C values (96%) in our healthcare system were calculated using the Friedewald equation. The remaining were quantified directly through homogeneous enzymatic assays.

Exposures were analysed both as continuous variables and categorical risk groups. Continuous values were modelled separately. For categorical analyses, we applied clinically relevant cutoffs aligned with prior studies.^13–15^ LDL-C was categorized using 1.8 mmol/L (70 mg/dL), a commonly used decision threshold/goal for very-high-risk ASCVD in European dyslipidaemia guidance and reflected in Swedish cardiovascular prevention recommendations during the study period. hsCRP was categorized using 2 mg/L, consistent with thresholds used in major cardiovascular trials and the residual inflammatory risk framework:

- *Low risk:* LDL-C < 1.8 mmol/L and hsCRP < 2 mg/L
- *High cholesterol risk (CR) alone:* LDL-C ≥ 1.8 mmol/L and hsCRP < 2 mg/L
- *High inflammatory risk (IR) alone:* LDL-C < 1.8 mmol/L and hsCRP ≥ 2 mg/L
- *Combined high cholesterol and inflammatory risk (CIR):* LDL-C ≥ 1.8 mmol/L and hsCRP ≥ 2 mg/L

In routine care settings, elevations in these two biomarkers may represent inadequate treatment response, undertreatment, unaddressed pathophysiology or all of the above.

### Study covariates

Study covariates were derived at the index date and included age, sex, comorbid conditions, ongoing medications, and laboratory tests (see Supplementary material online, *Table S3*). Medications were considered ongoing if dispensed within 6 months prior to the index date, based on data from the National Prescribed Drug Register, which captures all prescriptions filled in Swedish pharmacies. Laboratory covariates included outpatient serum/plasma creatinine, haemoglobin, and albumin or albuminuria measures. If unavailable at the index date, values were drawn from the prior 12 months. Missing laboratory values, reflecting tests not ordered during the ascertainment window, were handled by including a ‘missing’ category for each variable in adjusted models, given that missingness is not at random. Clinical diagnoses and medication data were complete. The National Prescribed Drug Register captures all dispensed prescriptions in Swedish pharmacies; absence of a dispensation record indicated non-use of that medication. This registry does not include, however, over-the-counter treatments and those dispensed directly at the hospital.

Estimated glomerular filtration rate (eGFR) was calculated with the CKD–Epidemiology Collaboration equation,^16^ and categorized as per KDIGO guidelines^17^ as: ≥60 and <60 mL/min per 1.73 m², the latter denoting moderate to severe CKD. Patients on maintenance dialysis or kidney transplantation (identified via linkage with the Swedish Renal Registry) were included in the CKD group. Albuminuria tests considered both urinary albumin to creatinine ratio (UACR, either on-spot urine sample, morning, evening, or 24-hour collection) and dipstick albuminuria/proteinuria, and were categorized as <30 mg/g, 30–300 mg/g, or >300 mg/g according to KDIGO thresholds.^17^

### Study outcomes

Outcomes included major adverse cardiovascular events (MACE: composite of cardiovascular death, or hospitalization for myocardial infarction or stroke), all-cause death, cardiovascular death, non-cardiovascular death, and hospitalization for heart failure. Event definitions are provided in Supplementary material online, *Table S4*. Outcomes were identified via linkage with regional health databases and the Swedish death registry, which captures vital status and cause of death for all residents. Follow-up extended from the index date until event occurrence, emigration, or 31 December 2021—whichever came first.

### Statistical analyses

Descriptive statistics are shown as median with first and third quartile (Q1-Q3), means with standard deviation (SD), or counts with percentages.

Cumulative incidence functions were estimated using the Aalen–Johansen estimator, treating death as a competing risk. Absolute risk differences (ARD) with 95% confidence intervals were calculated comparing each risk category to the low-risk reference group, with standard errors derived using the variance propagation method. Cause-specific Cox proportional hazards models were used to estimate hazard ratios (HRs) for associations between exposures and outcomes, accounting for the competing risk of death. These models estimate the association between the exposure and the outcome, treating death as a censoring event. We present both unadjusted models and models adjusted for potential confounders (age, sex, time since ASCVD, eGFR, albuminuria, comorbidities, undertaken procedures and ongoing medications as listed in *Table 1*). The proportional hazards assumption was assessed using Schoenfeld residuals and was not violated for the primary exposure variable (global test *P* > 0.05).

**Table 1:** Baseline characteristics of patients with ASCVD stratified by inflammatory and cholesterol risk categories

	Overall, *n* = 39 638	Low risk, *n* = 5351	High cholesterol risk, *n* = 13 636	High inflammatory risk, *n* = 4751	Combined high risk, *n* = 15 900	SMDs
**hsCRP**, mg/L; median [Q1–Q3]	2.0 [0.9, 4.1]	0.9 [0.9, 1.0]	0.9 [0.9, 1.0]	4.0 [2.7, 7.3]	4.0 [2.7, 7.1]	1.652
**LDL-C**, mmol/L; median [Q1–Q3]	2.30 [1.75, 3.10]	1.43 [1.20, 1.60]	2.60 [2.10, 3.25]	1.45 [1.20, 1.60]	2.70 [2.20, 3.50]	2.341
**Age,** years; mean [SD]	69 [12]	68 [11]	68 [12]	70 [11]	70 [12]	0.190
**Sex**, n(%)						
Men	24 027 (61%)	3939 (74%)	8035 (59%)	3217 (68%)	8836 (56%)	0.380
**Time since ASCVD**, *n* (%)						
<6 months	6088 (15%)	1166 (22%)	1888 (14%)	963 (20%)	2071 (13%)	0.233
6 months –<2 years	15 573 (39%)	2323 (43%)	5591 (41%)	1757 (37%)	5902 (37%)	0.132
2 years –<5 years	10 164 (26%)	1060 (20%)	3519 (26%)	1091 (23%)	4494 (28%)	0.198
≥5 years	7813 (20%)	802 (15%)	2638 (19%)	940 (20%)	3433 (22%)	0.169
**Haemoglobin**,mg/dL; mean [SD]	136 [16]	137 [15]	139 [14]	131 [17]	136 [16]	0.457
**Haemoglobin categories**, mg/dL, n (%)						
>150	6289 (16%)	900 (17%)	2400 (18%)	554 (12%)	2435 (15%)	0.165
131–150	18 726 (47%)	2665 (50%)	6836 (50%)	1975 (42%)	7250 (46%)	0.172
110–130	9936 (25%)	1350 (25%)	2812 (21%)	1599 (34%)	4175 (26%)	0.297
<110	2070 (5.2%)	250 (4.7%)	425 (3.1%)	490 (10%)	905 (5.7%)	0.306
Missing	2617 (6.6%)	186 (3.5%)	1163 (8.5%)	133 (2.8%)	1135 (7.1%)	0.253
**eGFR categories**, n (%)						
≥60 mL/min/1.73 m^2^	31 779 (80%)	4419 (83%)	11 566 (85%)	3435 (72%)	12 359 (78%)	0.312
30–59 mL/min/1.73 m^2^	6919 (17%)	837 (16%)	1922 (14%)	1110 (23%)	3050 (19%)	0.242
≤29 mL/min/1.73 m^2^	940 (2.4%)	95 (1.8%)	148 (1.1%)	206 (4.3%)	491 (3.1%)	0.206
**Albuminuria categories**, n (%)						
A1	22 932 (58%)	3297 (62%)	8141 (60%)	2588 (54%)	8906 (56%)	0.145
A2	5603 (14%)	684 (13%)	1573 (12%)	862 (18%)	2484 (16%)	0.188
A3	3551 (9.0%)	383 (7.2%)	894 (6.6%)	623 (13%)	1651 (10%)	0.227
missing	7552 (19%)	987 (18%)	3028 (22%)	678 (14%)	2859 (18%)	0.206
KDIGO CKD Stages, *n* (%)						
1–2	5968 (15%)	721 (13%)	1742 (13%)	862 (18%)	2643 (17%)	0.150
3a	4121 (10%)	536 (10%)	1224 (9.0%)	623 (13%)	1738 (11%)	0.134
3b	1776 (4.5%)	181 (3.4%)	415 (3.0%)	361 (7.6%)	819 (5.2%)	0.214
4/5	937 (2.4%)	93 (1.7%)	148 (1.1%)	205 (4.3%)	491 (3.1%)	0.205
No CKD	19 385 (49%)	2839 (53%)	7090 (52%)	2049 (43%)	7407 (47%)	0.199
Indeterminate	7451 (19%)	981 (18%)	3017 (22%)	651 (14%)	2802 (18%)	0.220
**Comorbid conditions**, n (%)						
Diabetes mellitus	10 932 (28%)	1679 (31%)	2679 (20%)	2023 (43%)	4551 (29%)	0.506
Hypertension	28 843 (73%)	3925 (73%)	9064 (66%)	3862 (81%)	11 992 (75%)	0.341
Myocardial infarction	11 612 (29%)	2391 (45%)	3457 (25%)	1917 (40%)	3847 (24%)	0.442
CABG	2505 (6.3%)	378 (7.1%)	704 (5.2%)	411 (8.7%)	1012 (6.4%)	0.139
PCI	11 128 (28%)	2510 (47%)	3561 (26%)	1783 (38%)	3274 (21%)	0.089
Angina	15 281 (39%)	1947 (36%)	5479 (40%)	1705 (36%)	6150 (39%)	0.574
Heart failure	7025 (18%)	945 (18%)	1710 (13%)	1249 (26%)	3121 (20%)	0.353
Stroke/TIA	12 725 (32%)	1448 (27%)	4460 (33%)	1498 (32%)	5319 (33%)	0.138
Peripheral vascular disease	4293 (11%)	360 (6.7%)	1156 (8.5%)	618 (13%)	2159 (14%)	0.225
Atrial fibrillation	7641 (19%)	927 (17%)	2138 (16%)	1216 (26%)	3360 (21%)	0.249
Rheumatoid diseases	5030 (13%)	589 (11%)	1236 (9.1%)	846 (18%)	2359 (15%)	0.260
Inflammatory bowel disease	636 (1.6%)	84 (1.6%)	190 (1.4%)	93 (2.0%)	269 (1.7%)	0.044
Chronic respiratory disease	7231 (18%)	843 (16%)	2054 (15%)	1000 (21%)	3334 (21%)	0.156
Recent cancer (3 years)	4547 (11%)	535 (10.0%)	1373 (10%)	656 (14%)	1983 (12%)	0.119
Recent anaemia (1 year)	8703 (22%)	1173 (22%)	2255 (17%)	1625 (34%)	3650 (23%)	0.419
Dyslipidaemia	38 219 (96%)	4717 (88%)	13 636 (100%)	3966 (83%)	15 900 (100%)	0.671
**Ongoing medications**, *n* (%)						
Antiplatelet	28 772 (73%)	4610 (86%)	9800 (72%)	3750 (79%)	10 612 (67%)	0.461
Aspirin	16 467 (42%)	2064 (39%)	5913 (43%)	1750 (37%)	6740 (42%)	0.133
P2Y12	2770 (7.0%)	413 (7.7%)	994 (7.3%)	399 (8.4%)	964 (6.1%)	0.089
DAPT	9457 (24%)	2128 (40%)	2865 (21%)	1592 (34%)	2872 (18%)	0.493
Other	78 (0.2%)	5 (<0.1%)	28 (0.2%)	9 (0.2%)	36 (0.2%)	0.031
ACEIs/ARBs	24 874 (63%)	3828 (72%)	7846 (58%)	3428 (72%)	9772 (61%)	0.311
MRAs	2563 (6.5%)	391 (7.3%)	627 (4.6%)	449 (9.5%)	1096 (6.9%)	0.190
β-blockers	24 204 (61%)	3715 (69%)	7648 (56%)	3411 (72%)	9430 (59%)	0.331
SGLT-2 inhibitors	576 (1.5%)	137 (2.6%)	108 (0.8%)	149 (3.1%)	182 (1.1%)	0.172
GLP-1 agonists	572 (1.4%)	131 (2.4%)	77 (0.6%)	174 (3.7%)	190 (1.2%)	0.224
Metformin	5433 (14%)	1001 (19%)	1332 (9.8%)	1000 (21%)	2100 (13%)	0.313
Diuretics	9549 (24%)	1097 (21%)	2391 (18%)	1590 (33%)	4471 (28%)	0.372
Calcium channel blockers	11 752 (30%)	1604 (30%)	3571 (26%)	1618 (34%)	4959 (31%)	0.171
Digoxin	888 (2.2%)	64 (1.2%)	190 (1.4%)	180 (3.8%)	454 (2.9%)	0.173
LLT (statins/PCSK-9i, ezetimibe)	24 238 (61%)	4380 (82%)	7975 (58%)	3616 (76%)	8267 (52%)	0.658
High-intensity LLT	9008 (37%)	2253 (51%)	2686 (34%)	1561 (43%)	2508 (30%)	0.438
Moderate-intensity LLT	13 588 (56%)	1928 (44%)	4718 (59%)	1843 (51%)	5099 (62%)	0.358
Low-intensity LLT	1642 (6.8%)	199 (4.5%)	571 (7.2%)	212 (5.9%)	660 (8.0%)	0.141
Fibrates, resins, and nicotinic acid	227 (0.6%)	17 (0.3%)	74 (0.5%)	19 (0.4%)	117 (0.7%)	0.059
Other blood pressure lowering drugs	611 (1.5%)	95 (1.8%)	135 (1.0%)	114 (2.4%)	267 (1.7%)	0.109
NSAIDs	4446 (11%)	423 (7.9%)	1482 (11%)	443 (9.3%)	2098 (13%)	0.174
Colchicine	66 (0.17%)	4 (0.07%)	13 (0.1%)	12 (0.25%)	37 (0.23%)	0.109
**Highest attained education**, *n* (%)						
Compulsory school	9335 (24%)	1150 (21%)	2740 (20%)	1300 (27%)	4145 (26%)	0.171
Secondary school	16 186 (41%)	2059 (38%)	5457 (40%)	1985 (42%)	6685 (42%)	0.073
University	13 136 (33%)	2030 (38%)	5200 (38%)	1301 (27%)	4605 (29%)	0.230
Missing	981 (2.5%)	112 (2.1%)	239 (1.8%)	165 (3.5%)	465 (2.9%)	0.109

ACEIs, Angiotensin-converting enzyme inhibitors; ARBs, Angiotensin receptor blockers; ASCVD, Atherosclerotic cardiovascular disease; CABG, Coronary artery bypass graft; CKD, Chronic kidney disease; DAPT, Dual antiplatelet therapy; eGFR, Estimated glomerular filtration rate; GLP-1, Glucagon-like peptide-1; hsCRP, High-sensitivity C-reactive protein; KDIGO, Kidney Disease: Improving Global Outcomes; LDL-C, Low-density lipoprotein cholesterol; LLT, Lipid-lowering therapy; MRA, Mineralocorticoid receptor antagonists; NSAIDs, Non-steroidal anti-inflammatory drugs; PCI, Percutaneous coronary intervention; PCSK-9i, Proprotein convertase subtilisin/kexin type 9 inhibitors; P2Y12, P2Y12 receptor inhibitor; SGLT-2, Sodium-glucose co-transporter-2; SMD, Standardized mean difference; TIA, Transient ischaemic attack.

Nonlinear relationships between hsCRP, LDL-C, and outcomes were explored using restricted cubic splines. Given the skewed distribution of hsCRP, we modelled its natural logarithm. Knots were placed at the 10^th^, 50^th^, and 90^th^ percentiles. For hsCRP, these percentiles occurred at 0.9, 2.0, and 9.0 mg/L, respectively. For LDL-C, these were at 1.4, 2.3, and 3.9 mmol/L. Non-linearity was tested using the Wald test, comparing the model with spline terms to a model assuming linearity (P for non-linearity).

Our primary subgroup analyses evaluated consistency of associations across eGFR strata (≥60 vs. < 60 mL/min/1.73 m²) and ongoing LLT use (on vs. off). Pre-specified subgroup analyses were also performed stratified by sex (men vs. women) and age (<65 vs. ≥ 65 years). Additionally, we stratified by calendar periods (pre-2016^18^ vs. 2016–2021^19,20^) to explore the possible impact of changes in European LDL-C guidelines, which introduced stricter lipid targets and newer LLT strategies. Interaction terms between risk categories and subgroup variables were tested, with *P* < 0.05 indicating significant heterogeneity.

Sensitivity analyses considered (i) excluding patients with baseline hsCRP > 10 mg/L and (ii) excluding events occurring within the first 6 or 12 months of follow-up to evaluate the impact of potential reverse causation bias (i.e. laboratory testing prompted by early disease signs).

## Results

### Study cohort selection

**Figure 1:**
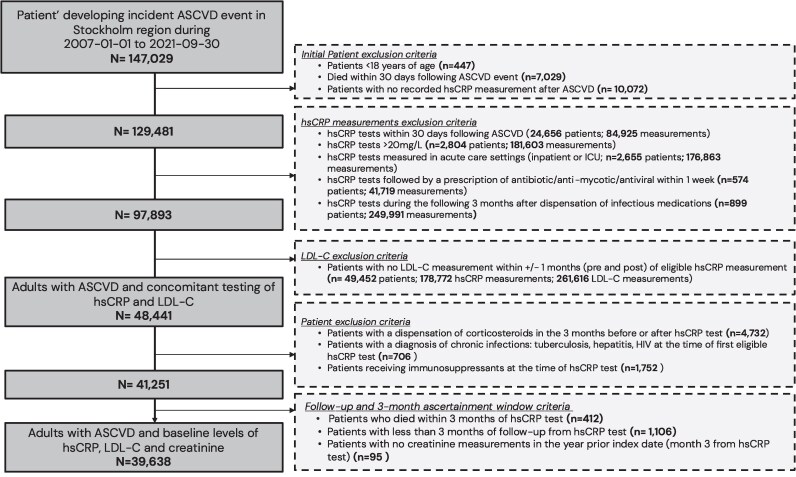
Patient selection flow chart. Abbreviations: ASCVD: Atherosclerotic cardiovascular disease; CRP: C-reactive protein; HIV: Human immunodeficiency virus; ICU: Intensive care unit; LDL-C: Low-density lipoprotein cholesterol.

A total of 39 638 adults with ASCVD met the inclusion criteria and were included in the analysis (*Figure 1*). The main exclusion criterion was the absence of concomitant monitoring of hsCRP and LDL-C, which halved the sample size. During the 3-month eligibility window used to define baseline hsCRP and LDL-C levels, there were 47 373 hsCRP and 44 726 LDL-C tests. Based on the geometric mean of these hsCRP and LDL-C levels, 5351 participants (13.5%) were classified as low risk, 13 636 (34.4%) as having CR, 4751 (12.0%) as having IR, and 15 900 (40.1%) as having CIR.

### Baseline characteristics

The mean age of the overall cohort was 69 (SD ±12) years, and 61% were men (*Table 1*). Characteristics varied substantially across risk groups. Patients with IR were older (mean age 70) and had the highest proportion of men (68%), followed by CR (59%) and CIR (56%). Comorbidities—including diabetes (43%), hypertension (81%), heart failure (26%), anaemia (34%), rheumatoid disease (18%), and atrial fibrillation (26%)—were most common among those with IR. In contrast, these conditions were less prevalent in the low-risk and CR groups.

Use of statins, other LLTs, and high-intensity LLT was highest in the low-risk group and lowest among those with CIR. CKD and albuminuria were also most common in the IR (27.3% and 31.2%, respectively) and CIR groups (22.1% and 26.1%) and least prevalent in patients with CR (15.1% and 18.2%).

Stratification by CKD status (see Supplementary material online, *Table S5*) showed that patients with eGFR < 60 mL/min/1.73 m² were older and had a higher burden of comorbidities. When stratified by LLT (see Supplementary material online, *Table S6*), median LDL-C levels for the IR and CIR groups were higher in patients without LLT, with minimal differences in hsCRP or age across all risk categories.

### Association between risk categories and clinical outcomes

During a median follow-up of 4.54 years [inter-quartile range (IQR) 2.04–7.68], there were 5349 MACE events, 7955 deaths (2088 cardiovascular-related), and 4285 hospitalizations for heart failure (see Supplementary material online, *Table S7*). Restricted cubic spline analyses demonstrated dose–response relationships for both hsCRP and LDL-C with respect to MACE (*P* for non-linearity = 0.83 and 0.34, respectively; Supplementary material online, *Figure S2*).

**Figure 2:**
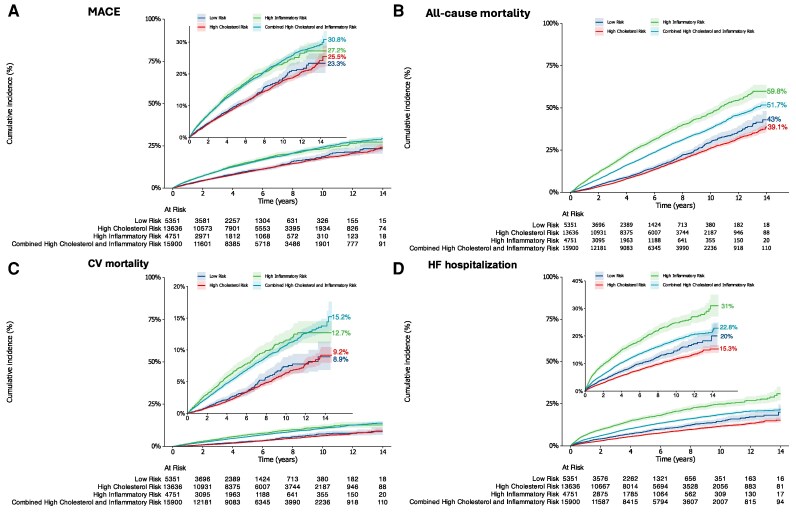
Cumulative incidence curves depicting the cumulative incidence of (*A*) MACE, and (*B*) all-cause mortality associated with high inflammatory and/or high cholesterol risk. Abbreviations: CV, Cardiovascular; HF, Heart failure; MACE, Major adverse cardiovascular events.

Compared with the low-risk group, individuals with IR and CIR had consistently higher incidence rates and 5-year absolute risks for all outcomes (*Table 2* and *Figure 2*). For example, the 5-year absolute risk of MACE was 10.0% [95% confidence interval (CI): 9.0–11.0%] in the low-risk group, compared with 15.2% (14.0%, 16.5%) in IR and 14.6% (14.0%, 15.2%) in CIR.

**Table 2:** Number of events, incidence rates, absolute risks and hazard ratios for adverse clinical outcomes associated with high inflammatory and/or high cholesterol risk

	No. of events/No. of patients	Incidence rate per 1000 person-years (95% CI)	5-year absolute risks (95% CI)	5-year absolute risk difference	Crude HR (95% CI)	Adj. HR (95% CI)^a^
**Major adverse cardiovascular events**						
Low risk	486/5351	22.5 (20.5–24.6)	10.0% (9.0%, 11.0%)	ref.	ref.	ref.
High cholesterol risk	1560/13 636	21.2 (20.1–22.2)	9.5% (9.0%, 10.1%)	−0.4 (−1.6 to +0.7)	0.96 (0.86–1.06)	1.12 (1.01–1.24)
High inflammatory risk	655/4751	36.1 (33.4–39.0)	15.2% (14.0%, 16.5%)	+5.2 (+3.6 to +6.8)	1.60 (1.42–1.80)	1.18 (1.05–1.33)
Combined high risk	2639/15 900	33.4 (32.1–34.7)	14.6% (14.0%, 15.2%)	+4.6 (+3.4 to +5.8)	1.50 (1.36–1.65)	1.39 (1.26–1.54)
**All-cause mortality**						
Low risk	649/5351	28.5 (26.4–30.8)	11.8% (10.7%, 12.9%)	ref.	ref.	ref.
High cholesterol risk	2095/13 636	26.9 (25.8–28.1)	10.5% (9.9%, 11.1%)	−1.2 (−2.5 to +0.0)	0.90 (0.83–0.99)	0.99 (0.91–1.09)
High inflammatory risk	1228/4751	63.7 (60.2–67.4)	27.2% (25.6%, 28.8%)	+15.4 (+13.5 to +17.4)	2.23 (2.03–2.45)	1.47 (1.34–1.62)
Combined high risk	3983/15 900	46.8 (45.4–48.3)	19.7% (19.0%, 20.5%)	+8.0 (+6.7 to +9.3)	1.59 (1.46–1.72)	1.27 (1.16–1.38)
**Heart failure hospitalization**						
Low risk	413/5351	19.0 (17.2–20.9)	8.5% (7.6%, 9.4%)	ref.	ref.	ref.
High cholesterol risk	1059/13 636	14.1 (13.3–15.0)	6.7% (6.3%, 7.2%)	−1.7 (−2.8 to −0.7)	0.78 (0.69–0.87)	1.00 (0.89–1.12)
High inflammatory risk	733/4751	41.2 (38.2–44.3)	16.7% (15.5%, 18.0%)	+8.2 (+6.7 to +9.8)	2.15 (1.91–2.43)	1.38 (1.22–1.56)
Combined high risk	2080/15 900	26.1 (25.0–27.2)	12.0% (11.4%, 12.6%)	+3.5 (+2.4 to +4.6)	1.42 (1.28–1.58)	1.21 (1.08–1.35)
**CV mortality**						
Low risk	155/5351	6.8 (5.8–8.0)	3.0% (2.4%, 3.6%)	ref.	ref.	ref.
High cholesterol risk	508/13 636	6.5 (6.0–7.1)	2.6% (2.3%, 2.9%)	−0.4 (−1.0 to +0.3)	0.93 (0.78–1.11)	1.04 (0.87–1.25)
High inflammatory risk	300/4751	15.6 (13.8–17.4)	6.9% (6.0%, 7.8%)	+3.9 (+2.9 to +5.0)	2.28 (1.88–2.77)	1.39 (1.14–1.70)
Combined high risk	1125/15 900	13.2 (12.5–14.0)	5.8% (5.4%, 6.2%)	+2.8 (+2.1 to +3.5)	1.90 (1.60–2.24)	1.44 (1.21–1.71)
**Non-CV mortality**						
Low risk	494/5351	21.7 (19.9–23.7)	8.8% (7.8%, 9.8%)	ref.	ref.	ref.
High cholesterol risk	1587/13 636	20.4 (19.4–21.4)	7.9% (7.4%, 8.5%)	−0.9 (−2.0 to +0.3)	0.90 (0.81–0.99)	0.98 (0.89–1.09)
High inflammatory risk	928/4751	48.1 (45.1–51.3)	20.3% (18.9%, 21.7%)	+11.5 (+9.8 to +13.2)	2.21 (1.99–2.47)	1.50 (1.35–1.68)
Combined high risk	2858/15 900	33.6 (32.4–34.9)	14.0% (13.4%, 14.6%)	+5.2 (+4.0 to +6.3)	1.49 (1.35–1.64)	1.21 (1.10–1.34)

Adj, Adjusted; CI, Confidence interval; CV, Cardiovascular; eGFR, Estimated glomerular filtration rate; ref, Reference; ASCVD, Atherosclerotic cardiovascular disease; HR, Hazard ratio; MI, Myocardial infarction; TIA, Transient ischaemic attack; NSAID, Nonsteroidal anti-inflammatory drug; SGLT-2i, Sodium-glucose cotransporter-2 inhibitor; PCSK9i, Proprotein convertase subtilisin/kexin type 9 inhibitor; LLT, Lipid-lowering therapy; MACE, Major adverse cardiovascular events.

^a^Adjusted for age, sex, time since ASCVD, eGFR (as continuous variable), albuminuria, comorbidities (diabetes mellitus, hypertension, chronic respiratory disease, cancer, MI, angina, heart failure, peripheral vascular disease, stroke/TIA, atrial fibrillation, and rheumatoid diseases), undertaken procedures (coronary artery bypass grafting and percutaneous coronary intervention), and ongoing medications (antiplatelet, NSAIDs, angiotensin-converting enzyme inhibitors/angiotensin receptor blockers, mineralocorticoid-receptor antagonists, β blocker, SGLT-2i, diuretics, calcium channel blockers, digoxin, lipid-lowering treatment [statins, PCSK9i, ezetimibe]).

In absolute terms, patients with high IR had the largest 5-year risk differences compared with low risk patients: +15.4% points (95% CI +13.5 to +17.4) for all-cause mortality, +8.2% points (+6.7 to +9.8) for heart failure hospitalization, +5.2% points (+3.6 to +6.8) for MACE, and +3.9% points (+2.9 to +5.0) for CV mortality. The CIR group showed intermediate absolute differences, with ARDs of +4.6% points (+3.4 to +5.8) for MACE and +8.0% points (+6.7 to +9.3) for all-cause mortality (*Table 2*). Notably, CR alone was not associated with increased absolute risk compared with low-risk patients.

In multivariable-adjusted models (*Table 2*), both the CIR and IR groups were significantly associated with elevated risks across all outcomes. Compared with the low-risk group, the CIR group had the highest relative risks for MACE (adjusted HR 1.39; 95% CI: 1.26–1.54) and cardiovascular mortality (aHR 1.44; 95% CI: 1.21–1.71), whereas the IR group was most strongly associated with all-cause mortality (aHR 1.47; 95% CI: 1.34–1.62), non-cardiovascular mortality (aHR 1.50; 95% CI: 1.35–1.68), and heart failure hospitalization (aHR 1.38; 95% CI: 1.22–1.56). In contrast, CR alone was associated with a modest increase in MACE risk (aHR 1.12; 95% CI: 1.01–1.24), but not with other outcomes.

Results were robust in the sensitivity analyses. Exclusion of individuals with CRP >10 mg/L modestly attenuated associations but did not alter the overall interpretation (see Supplementary material online, *Table S8*). Similarly, removing events within the first 6 or 12 months of follow-up had minimal impact on HR estimates, supporting low risk of reverse causation (see Supplementary material online, *Table S9*). In sensitivity analyses excluding patients with chronic inflammatory conditions (rheumatoid diseases and inflammatory bowel disease; *n* = 5551), associations between risk categories and outcomes remained consistent (see Supplementary material online, *Table S10*).

### Sub-group analysis by LLT use, CKD status, and calendar period

Spline analyses stratified by CKD status or LLT use showed similar associations between hsCRP and MACE (see Supplementary material online, *Figures S3* and *S4*). There were no differences in the association between LDL-C and MACE rates between individuals with and without CKD. In contrast, LDL-C associations with MACE were stronger and more linear among patients on-LLT compared to off-LLT.

**Figure 3:**
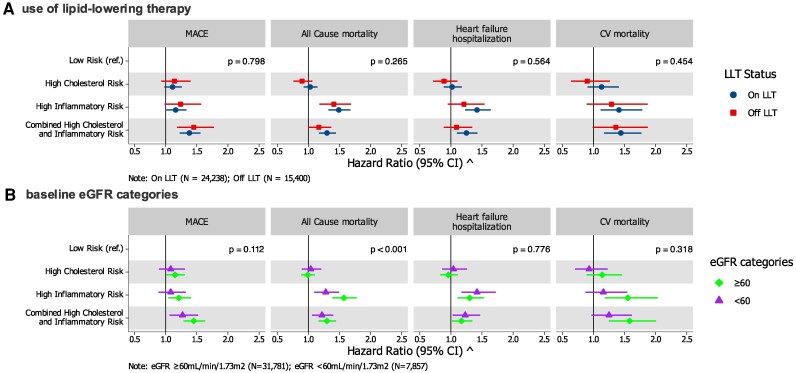
Subgroup analysis: association of high inflammatory and cholesterol risk categories with adverse cardiovascular outcomes, stratified by (*A*) use of LLT; (*B*) baseline eGFR categories. Abbreviations: Adj.: Adjusted; CI: Confidence interval; CV: Cardiovascular; eGFR: Estimated glomerular filtration rate; ref: Reference; ASCVD: Atherosclerotic cardiovascular disease; MI: Myocardial infarction; TIA: Transient ischaemic attack; NSAID: Nonsteroidal anti-inflammatory drug; SGLT-2i: Sodium-glucose cotransporter-2 inhibitor; PCSK9i: Proprotein convertase subtilisin/kexin type 9 inhibitor; LLT: Lipid-lowering therapy; MACE: Major adverse cardiovascular events. ^^^ adjusted for age, sex, time since ASCVD, eGFR (as continuous variable), albuminuria, comorbidities (diabetes mellitus, hypertension, chronic respiratory disease, cancer, MI, angina, heart failure, peripheral vascular disease, stroke/TIA, atrial fibrillation, and rheumatoid diseases), undertaken procedures (coronary artery bypass grafting and percutaneous coronary intervention), and ongoing medications (antiplatelet, NSAIDs, angiotensin-converting enzyme inhibitors/angiotensin receptor blockers, mineralocorticoid-receptor antagonists, β blocker, SGLT-2i, diuretics, calcium channel blockers, digoxin, lipid-lowering treatment [statins, PCSK9i, ezetimibe]). The *P* value reflects the test for interaction between the exposure (risk category) and the subgroup variable (e.g. LLT use or eGFR category) using a Wald test.

Associations between risk categories and clinical outcomes were directionally consistent across CKD and LLT subgroups (*Figure 3* and Supplementary material online, *Tables S11* and *S12*). Compared with the low-risk group, IR and CIR were associated with higher rates across all outcomes regardless of strata. Although high in both strata, the relative rates of all-cause mortality associated with IR were stronger in magnitude among those without CKD compared to those with CKD (p for interaction <0.001).

In subgroup analyses, associations between residual risk categories and outcomes were consistent across sex (*P*-interaction = 0.36 for MACE) and age groups, although effect sizes were generally larger in patients aged <65 years (*P*-interaction = 0.001 for MACE; Supplementary material online, *Tables S13* and *S14*). Analyses stratified by calendar period showed stronger associations between IR, CIR and all outcomes during 2016–2021 compared with earlier years (*P* for interaction ≤0.001; Supplementary material online, *Table S15*), possibly reflecting more contemporary treatment patterns or risk management strategies.

## Discussion

In this large cohort of adults with established ASCVD managed in routine care, we found that high inflammatory risk—either alone or in combination with high cholesterol risk—was associated with significantly higher rates of MACE, all-cause mortality, cardiovascular mortality, and heart failure hospitalization. These associations were robust across key subgroups, particularly among patients with CKD and those not receiving LLT. In contrast, high cholesterol risk alone was associated with a modestly increased risk of MACE but not with other adverse outcomes (Structured Graphical Abstract). These findings underscore the distinct and potentially complementary associations of inflammation and cholesterol with cardiovascular risk in contemporary clinical settings and support further consideration of inflammation monitoring in secondary prevention.

Our results extend observations previously derived mainly from clinical trials in statin-treated patients^3,21,22^ and prospective cohort studies.^23–25^ Evaluating real-world data is a valuable complement to these studies, as it captures more heterogeneous patient populations and care patterns, including variations in comorbidities, lifestyle, therapeutic adherence, and clinical decision-making. Notably, 39% of patients with ASCVD in our cohort were not using LLT at the time of assessment. This aligns with previous studies showing that 20–30% of patients with ASCVD discontinue LLT within the first year, with discontinuation increasing over time due to side effects, non-adherence, or clinical judgment.^26,27^

A recent pooled analysis by Ridker et al.^3^ of over 31 000 statin-treated patients from three major trials reported that individuals in the highest quartile of hsCRP had ∼30% higher risk of MACE and more than a 2.5-fold increase in cardiovascular mortality compared with those in the lowest quartile. In contrast, LDL-C levels in the highest quartile were not significantly associated with MACE (HR ∼1.07, *P* = 0.11) and had only a modest association with mortality. Some have criticized these findings as potentially confounded by the uniform provision of intensive statin therapy in trial settings.^28^ Our study provides a broader context by analysing routine care data. We observed that hsCRP associations with outcomes were largely independent of LLT use, whereas LDL-C associations were more pronounced among LLT users, reflecting true cholesterol risk not fully addressed by therapy. Among non-LLT users, associations were less clear as they represent both the risk associated with dyslipidaemia and reasons for not having treatment despite an indication. This aligns with UK Biobank analyses showing that biomarker concentrations, rather than LLT use alone, more strongly predicted cardiovascular risk.^23^

CKD represents a group of patients with particularly high risk due to complex lipid abnormalities, persistent inflammation, and elevated rates of ASCVD recurrence.^29^ In a *post hoc* analysis of 9151 patients from the CANTOS trial,^11^ increasing quartiles of hsCRP or IL-6 predicted MACE recurrence regardless of CKD status, whereas LDL-C and non-HDL-C did not (*n* = 1092 patients with CKD defined as eGFR <60 mL/min/1.73 m²). The authors concluded that residual inflammatory risk was the predominant predictor of events in this population. In contrast, our study, which included a substantially larger number of patients with CKD, found similar associations between hsCRP and LDL-C with MACE regardless of kidney function status. While both IR and CR were associated with MACE in patients with preserved kidney function, only the combination of both risks predicted MACE in those with CKD. We observed no significant interaction by kidney function, suggesting that differences in statistical power may explain the apparent subgroup variation. However, the possibility that CKD-specific mechanisms (e.g. oxidative stress, vascular ageing)^30,31^ contribute to these patterns cannot be excluded, nor can the potential limitation of standard biomarker thresholds to differentiate risks in this population (Mazhar *AJKD*, in press).

Our finding that isolated high cholesterol risk was only modestly associated with MACE and not with other outcomes is consistent with recent observational studies from Sweden,^32^ the USA,^25^ and China.^33^ This should not be interpreted as a lack of prognostic value of LDL-C—as our spline analyses demonstrate its continuous risk relationship—but rather as evidence that atherosclerotic disease progression involves multiple pathways. Our results support the hypothesis that targeting both lipid and inflammatory pathways may offer additive protection against ASCVD. Several trials^14,34,35^—have shown that combining LLT with anti-inflammatory therapy can yield incremental cardiovascular benefits.

Our study has several clinical implications. Current secondary prevention guidelines increasingly recognize the role of inflammation, recommending hsCRP measurement alongside LDL-C.^36^ Yet, this is not widely implemented in routine care.^37^ As an example, nearly half of the eligible population in our study lacked concurrent testing for both biomarkers and had to be excluded. Monitoring hsCRP together with LDL-C may enhance risk stratification, guide investigation of underlying causes of residual risk, and inform treatment intensification or lifestyle counselling. Our study can help reassure clinicians that measuring hsCRP along with LDL-C levels is clinically relevant if they wish to understand the risks faced by their patients. Limited adoption of this guideline recommendation may reflect clinical uncertainty about how to act on elevated hsCRP and the long-term safety and efficacy of available treatments. To date, colchicine remains the only approved anti-inflammatory therapy and pivotal trials were of relatively short duration (23–29 months).^14,35,38^ Several novel therapies targeting inflammation are currently under investigation and may open new avenues for treating inflammation in ASCVD.^39^

Strengths of this study include its large sample size, complete regional coverage, rich clinical data, and negligible loss to follow-up in a universal tax-funded healthcare setting, reducing selection bias from disparate access to healthcare or disaggregated data sources. However, several limitations warrant mention. The study was conducted in a single Swedish region with predominantly White patients, limiting generalizability. Outcomes were not adjudicated. Our requirement for concurrent hsCRP and LDL-C measurements may introduce selection towards patients receiving more intensive cardiovascular monitoring. However, this reflects the population for whom dual biomarker assessment should be clinically implemented and aligns with the analytical approach used in prior studies examining the intersection of inflammatory and lipid-related risk. Although hsCRP is a well-validated inflammatory marker, it is non-specific and may be elevated by non-cardiovascular or transient inflammatory conditions. Nonetheless, sensitivity analyses excluding values >10 mg/L or early events yielded consistent results. Residual confounding cannot be ruled out despite multivariable adjustment, particularly for unmeasured factors such as lifestyle or medication adherence. Lipoprotein(a), a causal cardiovascular risk factor, was not routinely measured in our healthcare; its contribution to residual risk, particularly in patients with elevated LDL-C, warrants investigation in future studies. Detailed measures of cardiac structure and function, including left ventricular ejection fraction, extent of coronary artery disease, and NYHA functional class, were not available in our database; however, we adjusted for clinical proxies of disease severity including heart failure history and revascularization procedures Additionally, while we attempted to exclude biomarker testing related to acute illness or chronic medications, indication bias remains possible. Our inclusion criteria, and mainly the selection of patients with concurrent testing of both LDL-C and hsCRP, may limit the generalizability of our findings. However, they are broadly consistent with prior randomized trials, in which such selection bias may have been less pronounced. Finally, as with all observational research, causality cannot be inferred.

## Conclusions

To conclude, in this large, real-world cohort of adults with ASCVD receiving routine care, hsCRP and LDL-C demonstrated distinct patterns of association with adverse outcomes when studied in combination. High inflammatory risk—alone or combined with high cholesterol risk—was independently associated with increased risk of cardiovascular events, mortality, and heart failure hospitalization. These findings were persistent regardless of LLT use and presence of CKD. Our study extends trial-based evidence into routine clinical practice and supports broader implementation of inflammation monitoring and treatment alongside lipid management in secondary ASCVD prevention.

## Supplementary Material

oeag023_Supplementary_Data

## Data Availability

The data underlying this article cannot be shared publicly due to the privacy of individuals who participated in the study. The data may be shared on reasonable request for academic research collaborations that fulfil GDPR as well as national and institutional ethics regulations and standards by contacting Prof. Juan-Jesus Carrero (juan.jesus.carrero@ki.se).

## Pre-registered clinical trial number

This study is not a clinical trial, but the SCREAM database is registered in ClinicalTrials.gov (NCT06239129).

## References

[1] Tsao CW, Aday AW, Almarzooq ZI, Alonso A, Beaton AZ, Bittencourt MS, Boehme AK, Buxton AE, Carson AP, Commodore-Mensah Y, Elkind MSV, Evenson KR, Eze-Nliam C, Ferguson JF, Generoso G, Ho JE, Kalani R, Khan SS, Kissela BM, Knutson KL, Levine DA, Lewis TT, Liu J, Loop MS, Ma J, Mussolino ME, Navaneethan SD, Perak AM, Poudel R, Rezk-Hanna M, Roth GA, Schroeder EB, Shah SH, Thacker EL, VanWagner LB, Virani SS, Voecks JH, Wang N-Y, Yaffe K, Martin SS. Heart disease and stroke statistics-2022 update: a report from the American Heart Association. Circulation 2022;145:e153–e639. 35078371 10.1161/CIR.0000000000001052

[2] Ference BA, Ginsberg HN, Graham I, Ray KK, Packard CJ, Bruckert E, Hegele RA, Krauss RM, Raal FJ, Schunkert H, Watts GF, Borén J, Fazio S, Horton JD, Masana L, Nicholls SJ, Nordestgaard BG, van de Sluis B, Taskinen M-R, Tokgözoğlu L, Landmesser U, Laufs U, Wiklund O, Stock JK, Chapman MJ, Catapano AL. Low-density lipoproteins cause atherosclerotic cardiovascular disease. 1. Evidence from genetic, epidemiologic, and clinical studies. A consensus statement from the European Atherosclerosis Society Consensus Panel. Eur Heart J 2017;38:2459–2472. 28444290 10.1093/eurheartj/ehx144 PMC5837225

[3] Ridker PM, Bhatt DL, Pradhan AD, Glynn RJ, MacFadyen JG, Nissen SE. Inflammation and cholesterol as predictors of cardiovascular events among patients receiving statin therapy: a collaborative analysis of three randomised trials. Lancet 2023;401:1293–1301. 36893777 10.1016/S0140-6736(23)00215-5

[4] Ridker PM, Everett BM, Thuren T, MacFadyen JG, Chang WH, Ballantyne C, Fonseca F, Nicolau J, Koenig W, Anker SD, Kastelein JJP, Cornel JH, Pais P, Pella D, Genest J, Cifkova R, Lorenzatti A, Forster T, Kobalava Z, Vida-Simiti L, Flather M, Shimokawa H, Ogawa H, Dellborg M, Rossi PRF, Troquay RPT, Libby P, Glynn RJ. Antiinflammatory therapy with canakinumab for atherosclerotic disease. N Engl J Med 2017;377:1119–1131. 28845751 10.1056/NEJMoa1707914

[5] Khan MS, Talha KM, Maqsood MH, Rymer JA, Borlaug BA, Docherty KF, Pandey A, Kahles F, Cikes M, Lam CSP, Ducharme A, Voors AA, Hernandez AF, Lincoff AM, Petrie MC, Ridker PM, Fudim M. Interleukin-6 and cardiovascular events in healthy adults: MESA. JACC Adv 2024;3:101063. 39077632 10.1016/j.jacadv.2024.101063 PMC11284704

[6] Fanola CL, Morrow DA, Cannon CP, Jarolim P, Lukas MA, Bode C, Hochman JS, Goodrich EL, Braunwald E, O’Donoghue ML. Interleukin-6 and the risk of adverse outcomes in patients after an acute coronary syndrome: observations from the SOLID-TIMI 52 (stabilization of plaque using darapladib—thrombolysis in myocardial infarction 52) Trial. J Am Heart Assoc 2017;6:e005637. 29066436 10.1161/JAHA.117.005637 PMC5721825

[7] Burger PM, Pradhan AD, Dorresteijn JAN, Koudstaal S, Teraa M, de Borst GJ, van der Meer MG, Mosterd A, Ridker PM, Visseren FLJ. C-Reactive Protein and risk of cardiovascular events and mortality in patients with various cardiovascular disease locations. Am J Cardiol 2023;197:13–23. 37218417 10.1016/j.amjcard.2023.03.025

[8] Held C, White HD, Stewart RAH, Budaj A, Cannon CP, Hochman JS, Koenig W, Siegbahn A, Steg PG, Soffer J, Weaver WD, Östlund O, Wallentin L; STABILITY Investigators. Inflammatory biomarkers interleukin-6 and C-reactive protein and outcomes in stable coronary heart disease: experiences from the STABILITY (stabilization of atherosclerotic plaque by initiation of darapladib therapy) Trial. J Am Heart Assoc 2017;6:e005077. 29066452 10.1161/JAHA.116.005077 PMC5721818

[9] C-reactive protein concentration and risk of coronary heart disease, stroke, and mortality: an individual participant meta-analysis. Lancet 2010; 375: 132–140. 20031199 10.1016/S0140-6736(09)61717-7 PMC3162187

[10] Mazhar F, Faucon A-L, Fu EL, Szummer KE, Mathisen J, Gerward S, Reuter SB, Marx N, Mehran R, Carrero J-J. Systemic inflammation and health outcomes in patients receiving treatment for atherosclerotic cardiovascular disease. Eur Heart J 2024;45:4719–4730. 39211962 10.1093/eurheartj/ehae557 PMC11578643

[11] Ridker PM, Tuttle KR, Perkovic V, Libby P, MacFadyen JG. Inflammation drives residual risk in chronic kidney disease: a CANTOS substudy. Eur Heart J 2022;43:4832–4844. 35943897 10.1093/eurheartj/ehac444

[12] Carrero JJ, Elinder CG. The Stockholm CREAtinine Measurements (SCREAM) project: fostering improvements in chronic kidney disease care. J Intern Med 2022;291:254–268. 35028991 10.1111/joim.13418

[13] Ridker PM . Residual inflammatory risk: addressing the obverse side of the atherosclerosis prevention coin. Eur Heart J 2016;37:1720–1722. 26908943 10.1093/eurheartj/ehw024

[14] Tardif J-C, Kouz S, Waters DD, Bertrand OF, Diaz R, Maggioni AP, Pinto FJ, Ibrahim R, Gamra H, Kiwan GS, Berry C, López-Sendón J, Ostadal P, Koenig W, Angoulvant D, Grégoire JC, Lavoie M-A, Dubé M-P, Rhainds D, Provencher M, Blondeau L, Orfanos A, L’Allier PL, Guertin M-C, Roubille F. Efficacy and safety of low-dose colchicine after myocardial infarction. N Engl J Med 2019;381:2497–2505. 31733140 10.1056/NEJMoa1912388

[15] Yang L, Yue Q, Fang F, Zhang Y, Liu P, Zhang Z, Wang G, Chen S, Wu S, Yang X. Effect of dual residual risk of cholesterol and inflammation on all-cause mortality in patients with cardiovascular disease. Cardiovasc Diabetol 2023;22:96. 37095492 10.1186/s12933-023-01826-3 PMC10127069

[16] Levey AS, Stevens LA, Schmid CH, Zhang Y, Castro AF, Feldman HI, Kusek JW, Eggers P, Van Lente F, Greene T, Coresh J. A new equation to estimate glomerular filtration rate. Ann Intern Med 2009;150:604–612. 19414839 10.7326/0003-4819-150-9-200905050-00006 PMC2763564

[17] Stevens PE, Levin A. Evaluation and management of chronic kidney disease: synopsis of the kidney disease: improving global outcomes 2012 clinical practice guideline. Ann Intern Med 2013;158:825–830. 23732715 10.7326/0003-4819-158-11-201306040-00007

[18] Reiner Ž, Catapano AL, De Backer G, Graham I, Taskinen M-R, Wiklund O, Agewall S, Alegria E, Chapman MJ, Durrington P, Erdine S, Halcox J, Hobbs R, Kjekshus J, Filardi PP, Riccardi G, Storey RF, Wood D, Bax J, Vahanian A, Auricchio A, Baumgartner H, Ceconi C, Dean V, Deaton C, Fagard R, Filippatos G, Funck-Brentano C, Hasdai D, Hobbs R, Hoes A, Kearney P, Knuuti J, Kolh P, McDonagh T, Moulin C, Poldermans D, Popescu BA, Reiner Z, Sechtem U, Sirnes PA, Tendera M, Torbicki A, Vardas P, Widimsky P, Windecker S, Reviewers: D, Funck-Brentano C, Poldermans D, Berkenboom G, De Graaf J, Descamps O, Gotcheva N, Griffith K, Guida GF, Gulec S, Henkin Y, Huber K, Kesaniemi YA, Lekakis J, Manolis AJ, Marques-Vidal P, Masana L, McMurray J, Mendes M, Pagava Z, Pedersen T, Prescott E, Rato Q, Rosano G, Sans S, Stalenhoef A, Tokgozoglu L, Viigimaa M, Wittekoek ME, Zamorano JL. ESC/EAS guidelines for the management of dyslipidaemias: the task force for the management of dyslipidaemias of the European Society of Cardiology (ESC) and the European Atherosclerosis Society (EAS). Eur Heart J 2011;32:1769–1818. 21712404 10.1093/eurheartj/ehr158

[19] Catapano AL, Graham I, De Backer G, Wiklund O, Chapman MJ, Drexel H, Hoes AW, Jennings CS, Landmesser U, Pedersen TR, Reiner Ž, Riccardi G, Taskinen M-R, Tokgozoglu L, Verschuren WMM, Vlachopoulos C, Wood DA, Zamorano JL. 2016 ESC/EAS guidelines for the management of dyslipidaemias. Eur Heart J 2016;37:2999–3058. 27567407 10.1093/eurheartj/ehw272

[20] Mach F, Baigent C, Catapano AL, Koskinas KC, Casula M, Badimon L, Chapman MJ, De Backer GG, Delgado V, Ference BA, Graham IM, Halliday A, Landmesser U, Mihaylova B, Pedersen TR, Riccardi G, Richter DJ, Sabatine MS, Taskinen M-R, Tokgozoglu L, Wiklund O, Mueller C, Drexel H, Aboyans V, Corsini A, Doehner W, Farnier M, Gigante B, Kayikcioglu M, Krstacic G, Lambrinou E, Lewis BS, Masip J, Moulin P, Petersen S, Petronio AS, Piepoli MF, Pintó X, Räber L, Ray KK, Reiner Ž, Riesen WF, Roffi M, Schmid J-P, Shlyakhto E, Simpson IA, Stroes E, Sudano I, Tselepis AD, Viigimaa M, Vindis C, Vonbank A, Vrablik M, Vrsalovic M, Zamorano JL, Collet J-P, Koskinas KC, Casula M, Badimon L, John Chapman M, De Backer GG, Delgado V, Ference BA, Graham IM, Halliday A, Landmesser U, Mihaylova B, Pedersen TR, Riccardi G, Richter DJ, Sabatine MS, Taskinen M-R, Tokgozoglu L, Wiklund O, Windecker S, Aboyans V, Baigent C, Collet J-P, Dean V, Delgado V, Fitzsimons D, Gale CP, Grobbee D, Halvorsen S, Hindricks G, Iung B, Jüni P, Katus HA, Landmesser U, Leclercq C, Lettino M, Lewis BS, Merkely B, Mueller C, Petersen S, Petronio AS, Richter DJ, Roffi M, Shlyakhto E, Simpson IA, Sousa-Uva M, Touyz RM, Nibouche D, Zelveian PH, Siostrzonek P, Najafov R, van de Borne P, Pojskic B, Postadzhiyan A, Kypris L, Špinar J, Larsen ML, Eldin HS, Viigimaa M, Strandberg TE, Ferrières J, Agladze R, Laufs U, Rallidis L, Bajnok L, Gudjónsson T, Maher V, Henkin Y, Gulizia MM, Mussagaliyeva A, Bajraktari G, Kerimkulova A, Latkovskis G, Hamoui O, Slapikas R, Visser L, Dingli P, Ivanov V, Boskovic A, Nazzi M, Visseren F, Mitevska I, Retterstøl K, Jankowski P, Fontes-Carvalho R, Gaita D, Ezhov M, Foscoli M, Giga V, Pella D, Fras Z, de Isla LP, Hagström E, Lehmann R, Abid L, Ozdogan O, Mitchenko O, Patel RS. 2019 ESC/EAS guidelines for the management of dyslipidaemias: lipid modification to reduce cardiovascular risk: the task force for the management of dyslipidaemias of the European Society of Cardiology (ESC) and European Atherosclerosis Society (EAS). Eur Heart J 2019;41:111–188. 10.1093/eurheartj/ehz455 31504418

[21] Ridker PM, Lei L, Louie MJ, Haddad T, Nicholls SJ, Lincoff AM, Libby P, Nissen S. Inflammation and cholesterol as predictors of cardiovascular events among 13 970 contemporary high-risk patients with statin intolerance. Circulation 2024;149:28–35. 37929602 10.1161/CIRCULATIONAHA.123.066213 PMC10752259

[22] Willeit P, Ridker PM, Nestel PJ, Simes J, Tonkin AM, Pedersen TR, Schwartz GG, Olsson AG, Colhoun HM, Kronenberg F, Drechsler C, Wanner C, Mora S, Lesogor A, Tsimikas S. Baseline and on-statin treatment lipoprotein(a) levels for prediction of cardiovascular events: individual patient-data meta-analysis of statin outcome trials. Lancet 2018;392:1311–1320. 30293769 10.1016/S0140-6736(18)31652-0

[23] Markus MRP, Ittermann T, Mariño Coronado J, Schipf S, Bahls M, Könemann S, Chamling B, Völzke H, Damasceno NRT, Santos RD, Felix SB, Templin C, Steinhagen-Thiessen E, Dörr M. LDL-cholesterol, lipoprotein(a) and high-sensitivity low-density lipoprotein cholesterol, lipoprotein(a) and high-sensitivity C-reactive protein are independent predictors of cardiovascular events. Eur Heart J 2025;46:3863–3874. 40320753 10.1093/eurheartj/ehaf281 PMC12517743

[24] Arnold N, Blaum C, Goßling A, Brunner FJ, Bay B, Ferrario MM, Brambilla P, Cesana G, Leoni V, Palmieri L, Donfrancesco C, Padró T, Andersson J, Jousilahti P, Ojeda F, Zeller T, Linneberg A, Söderberg S, Iacoviello L, Gianfagna F, Sans S, Veronesi G, Thorand B, Peters A, Tunstall-Pedoe H, Kee F, Salomaa V, Schnabel RB, Kuulasmaa K, Blankenberg S, Koenig W, Waldeyer C. C-reactive protein modifies lipoprotein(a)-related risk for coronary heart disease: the BiomarCaRE project. Eur Heart J 2024;45:1043–1054. 38240386 10.1093/eurheartj/ehad867

[25] Bay B, Tanner R, Gao M, Oliva A, Sartori S, Vogel B, Gitto M, Smith KF, Di Muro FM, Hooda A, Sweeny J, Krishnamoorthy P, Moreno P, Krishnan P, Dangas G, Kini A, Sharma SK, Mehran R. Residual cholesterol and inflammatory risk in statin-treated patients undergoing percutaneous coronary intervention. Eur Heart J 2025;46:3167–3177. 40208236 10.1093/eurheartj/ehaf196

[26] Mazhar F, Hjemdahl P, Clase CM, Johnell K, Jernberg T, Carrero JJ. Lipid-lowering treatment intensity, persistence, adherence and goal attainment in patients with coronary heart disease. Am Heart J 2022;251:78–90. 35654163 10.1016/j.ahj.2022.05.021

[27] Rezende Macedo do Nascimento RC, Mueller T, Godman B, MacBride Stewart S, Hurding S, de Assis Acurcio F, Guerra Junior AA, Alvares Teodoro J, Morton A, Bennie M, Kurdi A. Real-world evaluation of the impact of statin intensity on adherence and persistence to therapy: a Scottish population-based study. Br J Clin Pharmacol 2020;86:2349–2361. 32353163 10.1111/bcp.14333 PMC7688536

[28] Liuzzo G, Patrono C. Targeting residual cardiovascular risk in the statin era: cholesterol or inflammation? Eur Heart J 2023;44:1973–1975. 37138427 10.1093/eurheartj/ehad241

[29] Caturano A, Galiero R, Rocco M, Tagliaferri G, Piacevole A, Nilo D, Di Lorenzo G, Sardu C, Russo V, Vetrano E, Monda M, Marfella R, Rinaldi L, Sasso FC. The dual burden: exploring cardiovascular complications in chronic kidney disease. Biomolecules 2024;14:1393. 39595570 10.3390/biom14111393 PMC11591570

[30] Stenvinkel P, Wanner C, Metzger T, Heimbürger O, Mallamaci F, Tripepi G, Malatino L, Zoccali C. Inflammation and outcome in end-stage renal failure: does female gender constitute a survival advantage? Kidney Int 2002;62:1791–1798. 12371981 10.1046/j.1523-1755.2002.00637.x

[31] London GM . The clinical epidemiology of cardiovascular diseases in chronic kidney disease: cardiovascular disease in chronic renal failure: pathophysiologic aspects. Semin Dial 2003;16:85–94. 12641870 10.1046/j.1525-139x.2003.16023.x

[32] Ohm J, Hjemdahl P, Skoglund PH, Discacciati A, Sundström J, Hambraeus K, Jernberg T, Svensson P. Lipid levels achieved after a first myocardial infarction and the prediction of recurrent atherosclerotic cardiovascular disease. Int J Cardiol 2019;296:1–7. 31303394 10.1016/j.ijcard.2019.07.001

[33] Zhang H, Zhang C, Zhang Y, Tian T, Wang T, Chen J, Qian J, Hu F, Dou K, Qiao S, Wu Y, Guan C, Yang W, Song L. The role of residual inflammatory risk and LDL cholesterol in patients with in-stent restenosis undergoing percutaneous coronary intervention. J Clin Lipidol 2024;18:e746–e755. 39278780 10.1016/j.jacl.2024.05.009

[34] Ridker PM, MacFadyen JG, Thuren T, Libby P. Residual inflammatory risk associated with interleukin-18 and interleukin-6 after successful interleukin-1β inhibition with canakinumab: further rationale for the development of targeted anti-cytokine therapies for the treatment of atherothrombosis. Eur Heart J 2019;41:2153–2163. 10.1093/eurheartj/ehz542 31504417

[35] Nidorf SM, Fiolet ATL, Mosterd A, Eikelboom JW, Schut A, Opstal TSJ, The SHK, Xu X-F, Ireland MA, Lenderink T, Latchem D, Hoogslag P, Jerzewski A, Nierop P, Whelan A, Hendriks R, Swart H, Schaap J, Kuijper AFM, van Hessen MWJ, Saklani P, Tan I, Thompson AG, Morton A, Judkins C, Bax WA, Dirksen M, Alings M, Hankey GJ, Budgeon CA, Tijssen JGP, Cornel JH, Thompson PL. Colchicine in patients with chronic coronary disease. N Engl J Med 2020;383:1838–1847. 32865380 10.1056/NEJMoa2021372

[36] Vrints C, Andreotti F, Koskinas KC, Rossello X, Adamo M, Ainslie J, Banning AP, Budaj A, Buechel RR, Chiariello GA, Chieffo A, Christodorescu RM, Deaton C, Doenst T, Jones HW, Kunadian V, Mehilli J, Milojevic M, Piek JJ, Pugliese F, Rubboli A, Semb AG, Senior R, ten Berg JM, Van Belle E, Van Craenenbroeck EM, Vidal-Perez R, Winther S, Borger M, Gudmundsdóttir IJ, Knuuti J, Ahrens I, Böhm M, Buccheri S, Capodanno D, Christiansen EH, Collet J-P, Dickstein K, Eek C, Falk V, Henriksen PA, Ibanez B, James S, Kedev S, Køber L, Kyriakou M, Magavern EF, McInerney A, McEvoy JW, Mersha CO, Mihaylova B, Mindham R, Neubeck L, Neumann F-J, Nielsen JC, Paolisso P, Paradies V, Pasquet AA, Piepoli M, Prescott E, Rakisheva A, Rocca B, Ruel M, Sandner S, Saraste A, Szummer K, Vaartjes I, Wijns W, Windecker S, Witkowsky A, Zdrakovic M, Zeppenfeld K, Shuka N, Bouraghda MA, Hayrapetyan HG, Reinstadler SJ, Musayev O, De Pauw M, Kušljugić Z, Gelev V, Skoric B, Karakyriou M, Kovarnik T, Nielsen LH, Abdel-Aziz IS, Ainla T, Porela P, Benamer H, Nadaraia K, Richardt G, Papafaklis MI, Becker D, Gudmundsdóttir IJ, Wolak A, Riccio C, Zholdin BK, Elezi S, Abilova S, Mintale I, Allam B, Badarienė J, Pereira B, Dingli P, Revenco V, Bulatovic N, Benouna EGM, Dedic A, Mitevska I, Angel K, Bryniarski K, Luz AMC, Popescu BA, Bertelli L, Beleslin BD, Hudec M, Fras Z, Freixa-Pamias R, Holm A, Jeger R, Marjeh MYB, Hammami R, Aytekin V, Nesukay EG, Swanson N, Shek AB. 2024 ESC guidelines for the management of chronic coronary syndromes: developed by the task force for the management of chronic coronary syndromes of the European Society of Cardiology (ESC) endorsed by the European Association for Cardio-Thoracic Surgery (EACTS). Eur Heart J 2024;45:3415–3537.39210710

[37] Liuzzo G, Ridker PM. Universal screening for hsCRP in patients with atherosclerotic disease: a Major therapeutic opportunity. Eur Heart J 2024;45:4731–4733. 39211963 10.1093/eurheartj/ehae565

[38] Samuel M, Berry C, Dube M-P, Koenig W, López-Sendón J, Maggioni AP, Pinto FJ, Roubille F, Tardif J-C. Long-term trials of colchicine for secondary prevention of vascular events: a meta-analysis. Eur Heart J 2025;46:2552–2563. 40314333 10.1093/eurheartj/ehaf174 PMC12233006

[39] Potere N, Bonaventura A, Abbate A. Novel therapeutics and upcoming clinical trials targeting inflammation in cardiovascular diseases. Arterioscler Thromb Vasc Biol 2024;44:2371–2395. 39387118 10.1161/ATVBAHA.124.319980 PMC11602387

