# Supplementary material for: Inflammatory and cholesterol risks and rates of major cardiovascular events among patients with atherosclerotic cardiovascular disease in routine care
Source: Eur Heart J Open. 2026 Feb 17;6(2):oeag023. doi: 10.1093/ehjopen/oeag023 (PMC12962236; doi:10.1093/ehjopen/oeag023)
Supplement: oeag023_Supplementary_Data [file oeag023_supplementary_data.docx]

# **SUPPLEMENTARY MATERIAL**

## *Supplementary Methods*

**Detailed Study Design and Population**

This non-interventional, retrospective cohort study included adults (≥18 years old) with an incident clinical diagnosis of ASCVD (coronary, cerebrovascular, or peripheral artery disease) recorded between January 1, 2007, and September 30, 2021 (Supplemental Figure S1, Supplemental Table S1). The date of the first ASCVD diagnosis during this period defined the cohort entry date.

All subsequent hsCRP and LDL-C measurements recorded after cohort entry were extracted. To reflect chronic rather than acute inflammatory and lipid states, specific exclusion criteria were applied.

***Initial Patient Exclusion Criteria***

Patients were excluded if they:

- Were younger than 18 years at the time of the ASCVD diagnosis.
- Died within 30 days following their ASCVD diagnosis.
- Had no recorded hsCRP or LDL-C measurement after the ASCVD diagnosis.

***hsCRP Measurement Exclusion Criteria***

To ensure that hsCRP levels reflected underlying chronic inflammation rather than acute inflammatory events, we applied the following hsCRP-specific exclusions:

- hsCRP tests performed within 30 days after the ASCVD diagnosis.
- hsCRP tests taken during acute care settings, including inpatient stays or ICU visits (±1 day from admission/discharge), except for elective procedures.
- hsCRP values >20 mg/L, as these are likely indicative of acute inflammation.
- hsCRP tests followed by a prescription of antibiotics, antivirals, or antimycotics within one week, under the assumption that the test was performed to investigate an acute infection.
- hsCRP tests taken within 3 months of an infection diagnosis, as they may reflect monitoring of infection resolution rather than chronic inflammation.

**LDL-C Measurement exclusion criteria**

We included only outpatient tests performed as part of routine ASCVD monitoring. LDL-C values outside detection limits (<0.26 mmol/L and >10.3 mmol/L) were replaced by half the lower limit (0.13 mmol/L) or upper limit (10.3 mmol/L), respectively.

**Definition of Exposure Start Date**

We only considered hsCRP and LDL-C tests that measured on same day (as part of the same visit) or at most within 30 days of each other. The latest date of the pair defined the exposure start date. Only the earliest available paired measurement per patient was considered.

***Additional Patient Exclusion Criteria at the Exposure Start Date***

At the exposure start date, we further excluded patients with conditions or medications known to modify systemic inflammatory levels, including:

- Chronic infections, such as hepatitis, tuberculosis, or HIV.
- Recent cancer, defined as a cancer diagnosis in the 3 years prior to the exposure start date.
- Long-term use of corticosteroids or immunosuppressive drugs.

***Baseline hsCRP and LDL-C Definition and Ascertainment Window***

To ensure stable baseline measurements, high inflammatory risk and high cholesterol risk were defined based on all eligible outpatient tests performed within the 3-month ascertainment window following the exposure start date. The end of this ascertainment window was designated as the index date for the study.

***Follow-up and Additional Exclusion Criteria***

Patients were excluded if they:

- Died during the 3-month ascertainment window.
- Had less than 3 months of follow-up after the first eligible laboratory testing.
- Had no creatinine measurements in the year prior to the index date to estimate kidney function.

Following these criteria, the final cohort included ASCVD patients with stable, paired baseline hsCRP and LDL-C measurements and available measurements of creatinine to estimate kidney function.

## *Supplementary Tables*

**Table S1**: ICD-10 (Sweden) identification codes used for ASCVD

| Coronary event (acute myocardial infarction, angina) | ICD-10 I20, I21, I22, I23, I24, I25 |
| --- | --- |
| Cerebrovascular event (strokes and transient ischemic attack) | ICD-10 I63, I64, G459, I65, I66 ,167.2 |
| Peripheral artery disease | ICD-10 I73.9, I70 |
| Coronary revascularization | NOMESCO codes FNA-H |

ASCVD, atherosclerotic cardiovascular disease; ICD-10, International Classification of Diseases 10th Revision; NOMESCO, Nordic Medico-Statistical Committee classification of surgical procedures and (ATC) codes (World Health Organization).

**Table S2**: Exclusion criteria for hsCRP measurements and patients

|  | **ICD-10 and ATC codes (when needed)** |
| --- | --- |
| **Exclusion criteria for HSCRP tests** | |
| hsCRP tests performed within 30 days after the (most recent) ASCVD diagnosis |  |
| hsCRP tests followed by the dispensation of any antibiotic/antimycotic or antiviral within 7 days | J01, J02, J04, J05 |
| hsCRP tests during the 3 months following dispensation of infectious medications | J01, J02, J04, J05 |
| hsCRP tests during in-hospital stays and in ER (±1 day from admission/discharge) | Hospital measurements at admission of an elective surgery/procedures are accepted |
| A very high hsCRP (>20 mg/L) |  |
| **Exclusion criteria for patients with a first eligible hsCRP test** | |
| Patients receiving corticosteroids in the 3 months before or after the first eligible hsCRP test | H02 |
| Patients with a diagnosis of chronic infections (tuberculosis, hepatitis, HIV) at time of the first eligible hsCRP test | A15–A19, B15–B24 |
| Patients receiving immunosuppressants at time of the first eligible hsCRP test | L04 |

ASCVD, atherosclerotic cardiovascular disease; ATC, Anatomical Therapeutic Chemical; hsCRP, high sensitivity C-reactive protein; ER, emergency room; HIV, human immunodeficiency virus;
ICD-10, International Classification of Diseases 10th Revision (Sweden).

**Table S3**: Algorithms to define comorbid conditions and ongoing medications

| **Comorbid condition** | **ICD-10 or  NOMESCO codes** | **ATC code** |
| --- | --- | --- |
| Chronic infections (tuberculosis, hepatitis, and HIV) | A15–A19, B15–B24 |  |
| Diabetes | E10–E14 | A10 |
| Hypertension | I10–I15 |  |
| Chronic obstructive pulmonary disease | J40–J47 |  |
| Cancer (last 3 years) | Any code starting with C |  |
| Dementia | G30–G31 |  |
| Myocardial infarction | I21, I22 |  |
| Angina | I20 |  |
| Heart failure | I50, I110, I130-I132, K761 |  |
| Peripheral artery disease | I73.9, I70 |  |
| Stroke/TIA | I63-I66, I67.2, G459 |  |
| Atrial fibrillation | I48 |  |
| Coronary artery bypass graft | FNA–FNH except FNG |  |
| Percutaneous coronary intervention | FNG00–FNG06 |  |
| Inflammatory bowel diseases | K50–K51 |  |
| Rheumatoid diseases | L40, L93, M05, M06, M10, M140, M315, M353, M32, M34 |  |
| **Medication** |  | **ATC code** |
| Corticosteroids |  | H02 |
| Immunosuppressant |  | L04 |
| Antibiotics, antivirals, antimycotics |  | J01, J02, J05, D06AA, and D06AX |
| Antiplatelet |  | B01AC06, B01AC56, B01AC04, B01AC05, B01AC22, B01AC24, B01AC25, B01AC07, B01AC23, B01AC30 |
| Aspirin |  | B01AC06, B01AC56 |
| P2Y12i |  | B01AC04, B01AC05, B01AC22, B01AC24, B01AC25 |
| DAPT |  | B01AC30 or dispensation of both aspirin and P2Y12i in 6 months prior to index |
| Nonsteroidal anti-inflammatory drug |  | M01A |
| ACE inhibitors/renin–angiotensin system inhibitors |  | C09A–C09D |
| MRAs |  | C03DA01 |
| β-blockers |  | C07 |
| Diuretics |  | C03 |
| Calcium channel blockers |  | C08C–C08D |
| Digoxin |  | C01AA05 |
| Antidiabetics |  | A10 |
| LLT (statins, ezetimibe, PCSK9i) |  | C10AA, C10BA, C10AX09, C10AX13, C10AX14, C10AX17 |
| *High intensity LLT |  | ≥50% LDL-C reduction |
| *Moderate intensity LLT |  | 30–49% LDL-C reduction |
| *Low intensity LLT |  | <30% LDL-C reduction |
| Fibrates, resins, nicotinic acid |  | C10AB, C10AD |
| Other blood pressure medications |  | C02, C08E, C08G |

* LLT intensity was classified based on the expected percentage reduction in LDL-C for each statin dose, using thresholds defined in pivotal clinical trials and consistent with previously published methodology (Mazhar et al., JAHA 2022; https://doi.org/10.1161/JAHA.122.025813)

Abbreviations: ACE: Angiotensin-converting enzyme; ATC: Anatomical Therapeutic Chemical; DAPT: Dual antiplatelet therapy; HIV: Human immunodeficiency virus; ICD-10: International Classification of Diseases, 10th Revision; LLT: Lipid-lowering therapy; MRAs: Mineralocorticoid receptor antagonists; NOMESCO: Nordic Medico-Statistical Committee Classification of Surgical Procedures; P2Y12i: P2Y12 inhibitors; PCSK9i: Proprotein convertase subtilisin/kexin type 9 inhibitors; TIA: Transient ischemic attack.

**Table S4**: Algorithms to define study outcomes

| **Outcome** | **ICD-10 Codes** |
| --- | --- |
| Major adverse cardiac events (MACE) composite of: |  |
| Myocardial infarction | I21, I22, I23 |
| Ischemic stroke | I63 |
| CV mortality | Death attributed to  myocardial infarction: I21, I22, I252  congestive heart failure: I099, I110, I130, I132, I255, I420, I425-I429, I43, I50,  cerebrovascular disease: I70, I71, I731, I738, I739, I771, I790, I792, K551, K558, K559, Z958, Z959  peripheral arterial disease: G45-G46, H340, I60-I69  Atrial fibrillation: I48 |
| Heart failure hospitalization | I110, I130, I132, I50 |
| All-cause mortality | - |
| CVD death | Death attributed to  myocardial infarction: I21, I22, I252  congestive heart failure: I099, I110, I130, I132, I255, I420, I425-I429, I43, I50,  cerebrovascular disease: I70, I71, I731, I738, I739, I771, I790, I792, K551, K558, K559, Z958, Z959  peripheral arterial disease: G45-G46, H340, I60-I69  Atrial fibrillation: I48 |
| Non-CVD death | Any death not classified as CVD death |

Abbreviations: CV: Cardiovascular; CVD: Cardiovascular disease; ICD-10: International Classification of Diseases, Tenth Revision; MACE: Major adverse cardiac events.

**Table S5**: Baseline characteristics of patients with ASCVD stratified by high inflammatory and cholesterol risk categories and eGFR

|  | **Low risk,  N = 5,351** | | **High cholesterol risk, N = 13,636** | | **High inflammatory risk, N = 4,751** | | **Combined high risk, N = 15,900** | |
| --- | --- | --- | --- | --- | --- | --- | --- | --- |
|  | **eGFR ≥60** | **eGFR<60** | **eGFR ≥60** | **eGFR<60** | **eGFR ≥60** | **eGFR<60** | **eGFR ≥60** | **eGFR<60** |
|  | N = 4,419 | N = 932 | N = 11,566 | N = 2,070 | N = 3,435 | N = 1,316 | N = 12,359 | N = 3,541 |
| **hsCRP**, mg/L; median [Q1–Q3] | 0.9 [0.9, 1.0] | 1.0 [0.9, 1.0] | 0.9 [0.9, 1.0] | 0.9 [0.9, 1.0] | 4.0 [2.6, 7.0] | 4.8 [3.0, 8.0] | 4.0 [2.6, 7.0] | 4.9 [3.0, 8.4] |
| **LDL-C,** mmol/L; median [Q1–Q3] | 1.45 [1.20, 1.60] | 1.40 [1.20, 1.60] | 2.60 [2.10, 3.29] | 2.50 [2.10, 3.20] | 1.47 [1.20, 1.60] | 1.40 [1.20, 1.60] | 2.72 [2.20, 3.50] | 2.70 [2.20, 3.40] |
| **Age**, year; mean [SD] | 67 [11] | 77 [9] | 67 [11] | 78 [9] | 68 [11] | 77 [9] | 68 [11] | 78 [9] |
| **Sex**, n (%) |  |  |  |  |  |  |  |  |
| Men | 3,338 (76%) | 601 (64%) | 6,982 (60%) | 1,053 (51%) | 2,388 (70%) | 829 (63%) | 7,066 (57%) | 1,770 (50%) |
| **Time since ASCVD**, n (%) |  |  |  |  |  |  |  |  |
| <6 months | 1,028 (23%) | 138 (15%) | 1,673 (14%) | 215 (10%) | 766 (22%) | 197 (15%) | 1,712 (14%) | 359 (10%) |
| ≥6 months-<2 years | 1,946 (44%) | 377 (40%) | 4,759 (41%) | 832 (40%) | 1,308 (38%) | 449 (34%) | 4,626 (37%) | 1,276 (36%) |
| ≥2 years-<5 years | 828 (19%) | 232 (25%) | 2,977 (26%) | 542 (26%) | 755 (22%) | 336 (26%) | 3,433 (28%) | 1,061 (30%) |
| ≥5 years | 617 (14%) | 185 (20%) | 2,157 (19%) | 481 (23%) | 606 (18%) | 334 (25%) | 2,588 (21%) | 845 (24%) |
| **Hemoglobin**, mg/dL; mean [SD] | 138 [15] | 129 [16] | 140 [14] | 131 [16] | 134 [16] | 125 [17] | 138 [15] | 128 [17] |
| **Hemoglobin categories**, mg/dL, n (%) |  |  |  |  |  |  |  |  |
| >150 | 817 (18%) | 83 (8.9%) | 2,197 (19%) | 203 (9.8%) | 473 (14%) | 81 (6.2%) | 2,136 (17%) | 299 (8.4%) |
| >130-≤150 | 2,325 (53%) | 340 (36%) | 6,015 (52%) | 821 (40%) | 1,564 (46%) | 411 (31%) | 6,037 (49%) | 1,213 (34%) |
| ≥110-≤130 | 969 (22%) | 381 (41%) | 2,120 (18%) | 692 (33%) | 1,037 (30%) | 562 (43%) | 2,760 (22%) | 1,415 (40%) |
| <110 | 149 (3.4%) | 101 (11%) | 239 (2.1%) | 186 (9.0%) | 256 (7.5%) | 234 (18%) | 480 (3.9%) | 425 (12%) |
| Missing | 159 (3.6%) | 27 (2.9%) | 995 (8.6%) | 168 (8.1%) | 105 (3.1%) | 28 (2.1%) | 946 (7.7%) | 189 (5.3%) |
| **Albuminuria categories**, n (%) |  |  |  |  |  |  |  |  |
| A1 | 2,838 (64%) | 459 (49%) | 7,090 (61%) | 1,051 (51%) | 2,049 (60%) | 539 (41%) | 7,407 (60%) | 1,499 (42%) |
| A2 | 485 (11%) | 199 (21%) | 1,155 (10.0%) | 418 (20%) | 559 (16%) | 303 (23%) | 1,712 (14%) | 772 (22%) |
| A3 | 235 (5.3%) | 148 (16%) | 587 (5.1%) | 307 (15%) | 303 (8.8%) | 320 (24%) | 931 (7.5%) | 720 (20%) |
| Missing | 861 (19%) | 126 (14%) | 2,734 (24%) | 294 (14%) | 524 (15%) | 154 (12%) | 2,309 (19%) | 550 (16%) |
| KDIGO CKD Stages, n (%) |  |  |  |  |  |  |  |  |
| 01-Feb | 720 (16%) | 1 (0.1%) | 1,742 (15%) | 0 (0%) | 862 (25%) | 0 (0%) | 2,643 (21%) | 0 (0%) |
| 3a | 0 (0%) | 536 (58%) | 0 (0%) | 1,224 (59%) | 0 (0%) | 623 (47%) | 0 (0%) | 1,738 (49%) |
| 3b | 0 (0%) | 181 (19%) | 0 (0%) | 415 (20%) | 0 (0%) | 361 (27%) | 0 (0%) | 819 (23%) |
| 4/5 | 0 (0%) | 93 (10.0%) | 0 (0%) | 148 (7.1%) | 0 (0%) | 205 (16%) | 0 (0%) | 491 (14%) |
| No CKD | 2,838 (64%) | 1 (0.1%) | 7,090 (61%) | 0 (0%) | 2,049 (60%) | 0 (0%) | 7,407 (60%) | 0 (0%) |
| Indeterminate | 861 (19%) | 120 (13%) | 2,734 (24%) | 283 (14%) | 524 (15%) | 127 (9.7%) | 2,309 (19%) | 493 (14%) |
| **Comorbid conditions**, n (%) |  |  |  |  |  |  |  |  |
| Diabetes mellitus | 1,288 (29%) | 391 (42%) | 2,090 (18%) | 589 (28%) | 1,362 (40%) | 661 (50%) | 3,295 (27%) | 1,256 (35%) |
| Hypertension | 3,107 (70%) | 818 (88%) | 7,312 (63%) | 1,752 (85%) | 2,662 (77%) | 1,200 (91%) | 8,884 (72%) | 3,108 (88%) |
| Myocardial infarction | 2,018 (46%) | 373 (40%) | 2,927 (25%) | 530 (26%) | 1,419 (41%) | 498 (38%) | 2,969 (24%) | 878 (25%) |
| CABG | 322 (7.3%) | 56 (6.0%) | 601 (5.2%) | 103 (5.0%) | 330 (9.6%) | 81 (6.2%) | 836 (6.8%) | 176 (5.0%) |
| PCI | 2,141 (48%) | 369 (40%) | 3,089 (27%) | 472 (23%) | 1,353 (39%) | 430 (33%) | 2,615 (21%) | 659 (19%) |
| Angina | 1,633 (37%) | 314 (34%) | 4,694 (41%) | 785 (38%) | 1,283 (37%) | 422 (32%) | 4,897 (40%) | 1,253 (35%) |
| Heart failure | 633 (14%) | 312 (33%) | 1,177 (10%) | 533 (26%) | 703 (20%) | 546 (41%) | 1,843 (15%) | 1,278 (36%) |
| Stroke/TIA | 1,148 (26%) | 300 (32%) | 3,777 (33%) | 683 (33%) | 1,037 (30%) | 461 (35%) | 4,082 (33%) | 1,237 (35%) |
| Peripheral vascular disease | 256 (5.8%) | 104 (11%) | 882 (7.6%) | 274 (13%) | 368 (11%) | 250 (19%) | 1,486 (12%) | 673 (19%) |
| Atrial fibrillation | 662 (15%) | 265 (28%) | 1,612 (14%) | 526 (25%) | 738 (21%) | 478 (36%) | 2,235 (18%) | 1,125 (32%) |
| Rheumatoid diseases | 430 (9.7%) | 159 (17%) | 921 (8.0%) | 315 (15%) | 511 (15%) | 335 (25%) | 1,556 (13%) | 803 (23%) |
| Inflammatory bowel disease | 67 (1.5%) | 17 (1.8%) | 163 (1.4%) | 27 (1.3%) | 61 (1.8%) | 32 (2.4%) | 207 (1.7%) | 62 (1.8%) |
| Chronic respiratory disease | 673 (15%) | 170 (18%) | 1,713 (15%) | 341 (16%) | 691 (20%) | 309 (23%) | 2,506 (20%) | 828 (23%) |
| Recent cancer (3-years) | 419 (9.5%) | 116 (12%) | 1,072 (9.3%) | 301 (15%) | 434 (13%) | 222 (17%) | 1,419 (11%) | 564 (16%) |
| Recent anaemia (1-year) | 848 (19%) | 325 (35%) | 1,613 (14%) | 642 (31%) | 1,007 (29%) | 618 (47%) | 2,299 (19%) | 1,351 (38%) |
| Dyslipidemia | 3,896 (88%) | 821 (88%) | 11,566 (100%) | 2,070 (100%) | 2,895 (84%) | 1,071 (81%) | 12,359 (100%) | 3,541 (100%) |
| **Ongoing medications**, n (%) |  |  |  |  |  |  |  |  |
| Antiplatelet | 3,882 (88%) | 728 (78%) | 8,311 (72%) | 1,489 (72%) | 2,802 (82%) | 948 (72%) | 8,300 (67%) | 2,312 (65%) |
| Aspirin | 1,686 (38%) | 378 (41%) | 4,942 (43%) | 971 (47%) | 1,270 (37%) | 480 (36%) | 5,225 (42%) | 1,515 (43%) |
| P2Y12 | 335 (7.6%) | 78 (8.4%) | 850 (7.3%) | 144 (7.0%) | 283 (8.2%) | 116 (8.8%) | 765 (6.2%) | 199 (5.6%) |
| DAPT | 1,856 (42%) | 272 (29%) | 2,497 (22%) | 368 (18%) | 1,243 (36%) | 349 (27%) | 2,286 (18%) | 586 (17%) |
| Other | 5 (0.1%) | 0 (0%) | 22 (0.2%) | 6 (0.3%) | 6 (0.2%) | 3 (0.2%) | 24 (0.2%) | 12 (0.3%) |
| ACEIs/ARBs | 3,088 (70%) | 740 (79%) | 6,324 (55%) | 1,522 (74%) | 2,429 (71%) | 999 (76%) | 7,262 (59%) | 2,510 (71%) |
| MRAs | 274 (6.2%) | 117 (13%) | 414 (3.6%) | 213 (10%) | 254 (7.4%) | 195 (15%) | 648 (5.2%) | 448 (13%) |
| β-blockers | 3,033 (69%) | 682 (73%) | 6,275 (54%) | 1,373 (66%) | 2,419 (70%) | 992 (75%) | 7,024 (57%) | 2,406 (68%) |
| SGLT-2 inhibitors | 117 (2.6%) | 20 (2.1%) | 93 (0.8%) | 15 (0.7%) | 124 (3.6%) | 25 (1.9%) | 147 (1.2%) | 35 (1.0%) |
| GLP-1 agonists | 102 (2.3%) | 29 (3.1%) | 52 (0.4%) | 25 (1.2%) | 124 (3.6%) | 50 (3.8%) | 137 (1.1%) | 53 (1.5%) |
| Metformin | 850 (19%) | 151 (16%) | 1,139 (9.8%) | 193 (9.3%) | 838 (24%) | 162 (12%) | 1,773 (14%) | 327 (9.2%) |
| Diuretics | 706 (16%) | 391 (42%) | 1,556 (13%) | 835 (40%) | 860 (25%) | 730 (55%) | 2,645 (21%) | 1,826 (52%) |
| Calcium channel blockers | 1,226 (28%) | 378 (41%) | 2,822 (24%) | 749 (36%) | 1,077 (31%) | 541 (41%) | 3,552 (29%) | 1,407 (40%) |
| Digoxin | 46 (1.0%) | 18 (1.9%) | 127 (1.1%) | 63 (3.0%) | 107 (3.1%) | 73 (5.5%) | 282 (2.3%) | 172 (4.9%) |
| LLT (statins/PCSK-9i, ezetimibe) | 3,615 (82%) | 765 (82%) | 6,776 (59%) | 1,199 (58%) | 2,638 (77%) | 978 (74%) | 6,464 (52%) | 1,803 (51%) |
| High intensity LLT | 1,908 (53%) | 345 (45%) | 2,302 (34%) | 384 (32%) | 1,205 (46%) | 356 (36%) | 2,008 (31%) | 500 (28%) |
| Moderate intensity LLT | 1,549 (43%) | 379 (50%) | 4,016 (59%) | 702 (59%) | 1,289 (49%) | 554 (57%) | 3,943 (61%) | 1,156 (64%) |
| Low intensity LLT | 158 (4.4%) | 41 (5.4%) | 458 (6.8%) | 113 (9.4%) | 144 (5.5%) | 68 (7.0%) | 513 (7.9%) | 147 (8.2%) |
| Fibrates, resins, and nicotinic acid | 11 (0.2%) | 6 (0.6%) | 57 (0.5%) | 17 (0.8%) | 10 (0.3%) | 9 (0.7%) | 85 (0.7%) | 32 (0.9%) |
| Other blood pressure lowering drugs | 49 (1.1%) | 46 (4.9%) | 75 (0.6%) | 60 (2.9%) | 38 (1.1%) | 76 (5.8%) | 126 (1.0%) | 141 (4.0%) |
| NSAIDs | 360 (8.1%) | 63 (6.8%) | 1,319 (11%) | 163 (7.9%) | 345 (10%) | 98 (7.4%) | 1,759 (14%) | 339 (9.6%) |
| **Highest attained education**, n (%) |  |  |  |  |  |  |  |  |
| Compulsory school | 900 (20%) | 250 (27%) | 2,196 (19%) | 544 (26%) | 875 (25%) | 425 (32%) | 3,059 (25%) | 1,086 (31%) |
| Secondary school | 1,733 (39%) | 326 (35%) | 4,647 (40%) | 810 (39%) | 1,452 (42%) | 533 (41%) | 5,282 (43%) | 1,403 (40%) |
| University | 1,705 (39%) | 325 (35%) | 4,542 (39%) | 658 (32%) | 1,002 (29%) | 299 (23%) | 3,704 (30%) | 901 (25%) |
| Missing | 81 (1.8%) | 31 (3.3%) | 181 (1.6%) | 58 (2.8%) | 106 (3.1%) | 59 (4.5%) | 314 (2.5%) | 151 (4.3%) |

Abbreviations: ACEIs: Angiotensin-converting enzyme inhibitors; ARBs: Angiotensin receptor blockers; ASCVD: Atherosclerotic cardiovascular disease; CABG: Coronary artery bypass graft; CKD: Chronic kidney disease; DAPT: Dual antiplatelet therapy; eGFR: Estimated glomerular filtration rate; GLP-1: Glucagon-like peptide-1; hsCRP: High-sensitivity C-reactive protein; KDIGO: Kidney Disease: Improving Global Outcomes; LDL-C: Low-density lipoprotein cholesterol; LLT: Lipid-lowering therapy; MRA: Mineralocorticoid receptor antagonists; NSAIDs: Non-steroidal anti-inflammatory drugs; PCI: Percutaneous coronary intervention; PCSK-9i: Proprotein convertase subtilisin/kexin type 9 inhibitors; P2Y12: P2Y12 receptor inhibitor; SGLT-2: Sodium-glucose co-transporter-2; SMD: Standardized mean difference; TIA: Transient ischemic attack.

**Table S6**: Baseline characteristics of patients with ASCVD stratified by high inflammatory and cholesterol risk categories and LLT use

|  | **Low risk,** N = 5,351 | | **High cholesterol risk,** N = 13,636 | | **High inflammatory risk,** N = 4,751 | | **Combined high risk,** N = 15,900 | |
| --- | --- | --- | --- | --- | --- | --- | --- | --- |
|  | **Off LLT** N = 971 | **On LLT** N = 4,380 | **Off LLT** N = 5,661 | **On LLT** N = 7,975 | **Off LLT** N = 1,135 | **On LLT** N = 3,616 | **Off LLT** N = 7,633 | **On LLT** N = 8,267 |
| **hsCRP**, mg/L; median [Q1–Q3] | 0.9 [0.9, 1.0] | 0.9 [0.9, 1.0] | 0.9 [0.9, 1.0] | 0.9 [0.9, 1.0] | 4.9 [3.0, 9.0] | 4.0 [2.6, 7.0] | 4.2 [2.9, 8.0] | 4.0 [2.5, 7.0] |
| **LDL-C,** mmol/L; median [Q1–Q3] | 1.40 [1.21, 1.60] | 1.43 [1.20, 1.60] | 3.00 [2.40, 3.70] | 2.32 [2.00, 2.80] | 1.45 [1.20, 1.60] | 1.45 [1.20, 1.60] | 3.10 [2.50, 3.80] | 2.47 [2.10, 3.02] |
| **Age,**  years; mean [SD] | 69 [12] | 68 [11] | 68 [13] | 68 [11] | 71 [11] | 70 [11] | 70 [13] | 69 [11] |
| **Sex**, n(%) |  |  |  |  |  |  |  |  |
| Men | 678 (70%) | 3,261 (74%) | 2,919 (52%) | 5,116 (64%) | 729 (64%) | 2,488 (69%) | 3,840 (50%) | 4,996 (60%) |
| **Time since ASCVD**, n(%) |  |  |  |  |  |  |  |  |
| <6 months | 114 (12%) | 1,052 (24%) | 419 (7.4%) | 1,469 (18%) | 116 (10%) | 847 (23%) | 602 (7.9%) | 1,469 (18%) |
| ≥6 months-<2 years | 447 (46%) | 1,876 (43%) | 2,176 (38%) | 3,415 (43%) | 453 (40%) | 1,304 (36%) | 2,688 (35%) | 3,214 (39%) |
| ≥2 years-<5 years | 236 (24%) | 824 (19%) | 1,717 (30%) | 1,802 (23%) | 306 (27%) | 785 (22%) | 2,454 (32%) | 2,040 (25%) |
| ≥5 years | 174 (18%) | 628 (14%) | 1,349 (24%) | 1,289 (16%) | 260 (23%) | 680 (19%) | 1,889 (25%) | 1,544 (19%) |
| **Hemoglobin**, mg/dL; mean [SD] | 136 [16] | 137 [15] | 138 [14] | 139 [14] | 130 [18] | 132 [17] | 135 [16] | 136 [16] |
| **Hemoglobin categories**, mg/dL, n(%) |  |  |  |  |  |  |  |  |
| >150 | 171 (18%) | 729 (17%) | 945 (17%) | 1,455 (18%) | 120 (11%) | 434 (12%) | 1,121 (15%) | 1,314 (16%) |
| >130-≤150 | 428 (44%) | 2,237 (51%) | 2,748 (49%) | 4,088 (51%) | 435 (38%) | 1,540 (43%) | 3,415 (45%) | 3,835 (46%) |
| ≥110-≤130 | 264 (27%) | 1,086 (25%) | 1,238 (22%) | 1,574 (20%) | 395 (35%) | 1,204 (33%) | 2,033 (27%) | 2,142 (26%) |
| <110 | 56 (5.8%) | 194 (4.4%) | 172 (3.0%) | 253 (3.2%) | 148 (13%) | 342 (9.5%) | 453 (5.9%) | 452 (5.5%) |
| Missing | 52 (5.4%) | 134 (3.1%) | 558 (9.9%) | 605 (7.6%) | 37 (3.3%) | 96 (2.7%) | 611 (8.0%) | 524 (6.3%) |
| **eGFR categories**, n(%) |  |  |  |  |  |  |  |  |
| ≥60mL/min/1.73 m^2^ | 804 (83%) | 3,615 (83%) | 4,790 (85%) | 6,776 (85%) | 797 (70%) | 2,638 (73%) | 5,895 (77%) | 6,464 (78%) |
| ≥30-≤59mL/min/1.73 m^2^ | 140 (14%) | 697 (16%) | 797 (14%) | 1,125 (14%) | 280 (25%) | 830 (23%) | 1,468 (19%) | 1,582 (19%) |
| ≤29mL/min/1.73 m^2^ | 27 (2.8%) | 68 (1.6%) | 74 (1.3%) | 74 (0.9%) | 58 (5.1%) | 148 (4.1%) | 270 (3.5%) | 221 (2.7%) |
| **Albuminuria categories**, n(%) |  |  |  |  |  |  |  |  |
| A1 | 568 (58%) | 2,729 (62%) | 3,240 (57%) | 4,901 (61%) | 592 (52%) | 1,996 (55%) | 4,109 (54%) | 4,797 (58%) |
| A2 | 111 (11%) | 573 (13%) | 662 (12%) | 911 (11%) | 216 (19%) | 646 (18%) | 1,153 (15%) | 1,331 (16%) |
| A3 | 91 (9.4%) | 292 (6.7%) | 353 (6.2%) | 541 (6.8%) | 162 (14%) | 461 (13%) | 780 (10%) | 871 (11%) |
| Missing | 201 (21%) | 786 (18%) | 1,406 (25%) | 1,622 (20%) | 165 (15%) | 513 (14%) | 1,591 (21%) | 1,268 (15%) |
| KDIGO CKD Stages, n(%) |  |  |  |  |  |  |  |  |
| 1-2 | 138 (14%) | 583 (13%) | 721 (13%) | 1,021 (13%) | 220 (19%) | 642 (18%) | 1,215 (16%) | 1,428 (17%) |
| 3a | 82 (8.4%) | 454 (10%) | 504 (8.9%) | 720 (9.0%) | 151 (13%) | 472 (13%) | 826 (11%) | 912 (11%) |
| 3b | 28 (2.9%) | 153 (3.5%) | 165 (2.9%) | 250 (3.1%) | 86 (7.6%) | 275 (7.6%) | 373 (4.9%) | 446 (5.4%) |
| 4/5 | 27 (2.8%) | 66 (1.5%) | 74 (1.3%) | 74 (0.9%) | 58 (5.1%) | 147 (4.1%) | 270 (3.5%) | 221 (2.7%) |
| No CKD | 496 (51%) | 2,343 (53%) | 2,797 (49%) | 4,293 (54%) | 464 (41%) | 1,585 (44%) | 3,392 (44%) | 4,015 (49%) |
| Indeterminate | 200 (21%) | 781 (18%) | 1,400 (25%) | 1,617 (20%) | 156 (14%) | 495 (14%) | 1,557 (20%) | 1,245 (15%) |
| **Comorbid conditions**, n(%) |  |  |  |  |  |  |  |  |
| Diabetes mellitus | 294 (30%) | 1,385 (32%) | 943 (17%) | 1,736 (22%) | 474 (42%) | 1,549 (43%) | 1,978 (26%) | 2,573 (31%) |
| Hypertension | 695 (72%) | 3,230 (74%) | 3,509 (62%) | 5,555 (70%) | 940 (83%) | 2,922 (81%) | 5,537 (73%) | 6,455 (78%) |
| Myocardial infarction | 331 (34%) | 2,060 (47%) | 895 (16%) | 2,562 (32%) | 351 (31%) | 1,566 (43%) | 1,226 (16%) | 2,621 (32%) |
| CABG | 60 (6.2%) | 318 (7.3%) | 131 (2.3%) | 573 (7.2%) | 75 (6.6%) | 336 (9.3%) | 194 (2.5%) | 818 (9.9%) |
| PCI | 326 (34%) | 2,184 (50%) | 720 (13%) | 2,841 (36%) | 284 (25%) | 1,499 (41%) | 825 (11%) | 2,449 (30%) |
| Angina | 353 (36%) | 1,594 (36%) | 2,245 (40%) | 3,234 (41%) | 400 (35%) | 1,305 (36%) | 2,928 (38%) | 3,222 (39%) |
| Heart failure | 164 (17%) | 781 (18%) | 638 (11%) | 1,072 (13%) | 319 (28%) | 930 (26%) | 1,498 (20%) | 1,623 (20%) |
| Stroke/TIA | 317 (33%) | 1,131 (26%) | 2,035 (36%) | 2,425 (30%) | 412 (36%) | 1,086 (30%) | 2,691 (35%) | 2,628 (32%) |
| Peripheral vascular disease | 91 (9.4%) | 269 (6.1%) | 566 (10.0%) | 590 (7.4%) | 166 (15%) | 452 (13%) | 1,072 (14%) | 1,087 (13%) |
| Atrial fibrillation | 179 (18%) | 748 (17%) | 954 (17%) | 1,184 (15%) | 337 (30%) | 879 (24%) | 1,721 (23%) | 1,639 (20%) |
| Rheumatoid diseases | 111 (11%) | 478 (11%) | 520 (9.2%) | 716 (9.0%) | 195 (17%) | 651 (18%) | 1,145 (15%) | 1,214 (15%) |
| Inflammatory bowel disease | 23 (2.4%) | 61 (1.4%) | 85 (1.5%) | 105 (1.3%) | 20 (1.8%) | 73 (2.0%) | 129 (1.7%) | 140 (1.7%) |
| Chronic respiratory disease | 169 (17%) | 674 (15%) | 929 (16%) | 1,125 (14%) | 256 (23%) | 744 (21%) | 1,716 (22%) | 1,618 (20%) |
| Recent cancer (3-years) | 101 (10%) | 434 (9.9%) | 584 (10%) | 789 (9.9%) | 188 (17%) | 468 (13%) | 984 (13%) | 999 (12%) |
| Recent anaemia (1-year) | 214 (22%) | 959 (22%) | 950 (17%) | 1,305 (16%) | 424 (37%) | 1,201 (33%) | 1,726 (23%) | 1,924 (23%) |
| Dyslipidemia | 337 (35%) | 4,380 (100%) | 5,661 (100%) | 7,975 (100%) | 350 (31%) | 3,616 (100%) | 7,633 (100%) | 8,267 (100%) |
| **Ongoing medications**, n(%) |  |  |  |  |  |  |  |  |
| Antiplatelet | 726 (75%) | 3,884 (89%) | 3,030 (54%) | 6,770 (85%) | 766 (67%) | 2,984 (83%) | 3,939 (52%) | 6,673 (81%) |
| Aspirin | 410 (42%) | 1,654 (38%) | 2,207 (39%) | 3,706 (46%) | 456 (40%) | 1,294 (36%) | 2,967 (39%) | 3,773 (46%) |
| P2Y12 | 76 (7.8%) | 337 (7.7%) | 314 (5.5%) | 680 (8.5%) | 90 (7.9%) | 309 (8.5%) | 340 (4.5%) | 624 (7.5%) |
| DAPT | 239 (25%) | 1,889 (43%) | 496 (8.8%) | 2,369 (30%) | 219 (19%) | 1,373 (38%) | 613 (8.0%) | 2,259 (27%) |
| Other | 1 (0.1%) | 4 (<0.1%) | 13 (0.2%) | 15 (0.2%) | 1 (<0.1%) | 8 (0.2%) | 19 (0.2%) | 17 (0.2%) |
| ACEIs/ARBs | 594 (61%) | 3,234 (74%) | 2,613 (46%) | 5,233 (66%) | 714 (63%) | 2,714 (75%) | 3,995 (52%) | 5,777 (70%) |
| MRAs | 53 (5.5%) | 338 (7.7%) | 237 (4.2%) | 390 (4.9%) | 106 (9.3%) | 343 (9.5%) | 496 (6.5%) | 600 (7.3%) |
| β-blockers | 567 (58%) | 3,148 (72%) | 2,454 (43%) | 5,194 (65%) | 731 (64%) | 2,680 (74%) | 3,771 (49%) | 5,659 (68%) |
| SGLT-2 inhibitors | 21 (2.2%) | 116 (2.6%) | 30 (0.5%) | 78 (1.0%) | 24 (2.1%) | 125 (3.5%) | 54 (0.7%) | 128 (1.5%) |
| GLP-1 agonists | 19 (2.0%) | 112 (2.6%) | 24 (0.4%) | 53 (0.7%) | 30 (2.6%) | 144 (4.0%) | 79 (1.0%) | 111 (1.3%) |
| Metformin | 158 (16%) | 843 (19%) | 414 (7.3%) | 918 (12%) | 217 (19%) | 783 (22%) | 786 (10%) | 1,314 (16%) |
| Diuretics | 196 (20%) | 901 (21%) | 980 (17%) | 1,411 (18%) | 359 (32%) | 1,231 (34%) | 2,083 (27%) | 2,388 (29%) |
| Calcium channel blockers | 268 (28%) | 1,336 (31%) | 1,321 (23%) | 2,250 (28%) | 340 (30%) | 1,278 (35%) | 2,100 (28%) | 2,859 (35%) |
| Digoxin | 19 (2.0%) | 45 (1.0%) | 92 (1.6%) | 98 (1.2%) | 53 (4.7%) | 127 (3.5%) | 262 (3.4%) | 192 (2.3%) |
| LLT (statins/PCSK-9i, ezetimibe) | 0 (0%) | 4,380 (100%) | 0 (0%) | 7,975 (100%) | 0 (0%) | 3,616 (100%) | 0 (0%) | 8,267 (100%) |
| High intensity LLT | 0 (NA%) | 2,253 (51%) | 0 (NA%) | 2,686 (34%) | 0 (NA%) | 1,561 (43%) | 0 (NA%) | 2,508 (30%) |
| Moderate intensity LLT | 0 (NA%) | 1,928 (44%) | 0 (NA%) | 4,718 (59%) | 0 (NA%) | 1,843 (51%) | 0 (NA%) | 5,099 (62%) |
| Low intensity LLT | 0 (NA%) | 199 (4.5%) | 0 (NA%) | 571 (7.2%) | 0 (NA%) | 212 (5.9%) | 0 (NA%) | 660 (8.0%) |
| Fibrates, resins, and nicotinic acid | 6 (0.6%) | 11 (0.3%) | 34 (0.6%) | 40 (0.5%) | 6 (0.5%) | 13 (0.4%) | 65 (0.9%) | 52 (0.6%) |
| Other blood pressure lowering drugs | 17 (1.8%) | 78 (1.8%) | 62 (1.1%) | 73 (0.9%) | 27 (2.4%) | 87 (2.4%) | 114 (1.5%) | 153 (1.9%) |
| NSAIDs | 97 (10.0%) | 326 (7.4%) | 660 (12%) | 822 (10%) | 117 (10%) | 326 (9.0%) | 1,039 (14%) | 1,059 (13%) |
| **Highest attained education**, n(%) |  |  |  |  |  |  |  |  |
| Compulsory school | 206 (21%) | 944 (22%) | 1,112 (20%) | 1,628 (20%) | 304 (27%) | 996 (28%) | 1,990 (26%) | 2,155 (26%) |
| Secondary school | 375 (39%) | 1,684 (38%) | 2,252 (40%) | 3,205 (40%) | 513 (45%) | 1,472 (41%) | 3,167 (41%) | 3,518 (43%) |
| University | 361 (37%) | 1,669 (38%) | 2,189 (39%) | 3,011 (38%) | 277 (24%) | 1,024 (28%) | 2,235 (29%) | 2,370 (29%) |
| Missing | 29 (3.0%) | 83 (1.9%) | 108 (1.9%) | 131 (1.6%) | 41 (3.6%) | 124 (3.4%) | 241 (3.2%) | 224 (2.7%) |

Abbreviations: ACEIs: Angiotensin-converting enzyme inhibitors; ARBs: Angiotensin receptor blockers; ASCVD: Atherosclerotic cardiovascular disease; CABG: Coronary artery bypass graft; CKD: Chronic kidney disease; DAPT: Dual antiplatelet therapy; eGFR: Estimated glomerular filtration rate; GLP-1: Glucagon-like peptide-1; hsCRP: High-sensitivity C-reactive protein; KDIGO: Kidney Disease: Improving Global Outcomes; LDL-C: Low-density lipoprotein cholesterol; LLT: Lipid-lowering therapy; MRA: Mineralocorticoid receptor antagonists; NSAIDs: Non-steroidal anti-inflammatory drugs; PCI: Percutaneous coronary intervention; PCSK-9i: Proprotein convertase subtilisin/kexin type 9 inhibitors; P2Y12: P2Y12 receptor inhibitor; SGLT-2: Sodium-glucose co-transporter-2; SMD: Standardized mean difference; TIA: Transient ischemic attack.

**Table S7**: Count of individual components of each outcome

| **Endpoint** | **number of events** | **competing event type** | **competing event count** |
| --- | --- | --- | --- |
| MACE | 5349 | non cv death | 4880 |
| MI | 2194 |  |  |
| Stroke | 2043 |  |  |
| CV mortality | 1103 |  |  |
| All-cause mortality | 7955 | none |  |
| Heart failure hospitalization | 4285 | All Cause mortality | 5557 |
| MI | 2319 | non MI death | 6832 |
| Stroke | 2166 | non stroke death | 6953 |
| CV mortality | 2088 | non cv death | 5867 |

Abbreviations: CV: Cardiovascular; CVD: Cardiovascular disease; ICD-10: International Classification of Diseases, Tenth Revision; MACE: Major adverse cardiac events.

**Table S8**. Sensitivity analysis: number of events and hazard ratios for the risk of adverse outcomes associated with baseline hsCRP categories after exclusion of patients with a baseline hsCRP>10 mg/L.

|  | |  | | **After excluding 2944 patients with hsCRP>10 mg/L** | | | |
| --- | --- | --- | --- | --- | --- | --- | --- |
|  | | **Original analysis**  **(for comparison)**  **Adj. HR (95% CI)^a^** | | **Number of events/**  **number of patients** | | **Adj. HR (95% CI)^a^** | |
| **MACE** |  | |  | |  | |  |
| Low risk | | ref. | | 486/5351 | | ref. | |
| High cholesterol risk | | 1.12 (1.01-1.24) | | 1560/13636 | | 1.11 (1.00-1.24) | |
| High inflammatory risk | | 1.18 (1.05-1.33) | | 495/4004 | | 1.10 (0.97-1.24) | |
| Combined high risk | | 1.39 (1.26-1.54) | | 2161/13703 | | 1.34 (1.21-1.48) | |
| **All-cause mortality** | |  | |  | |  | |
| Low risk | | ref. | | 649/5351 | | ref. | |
| High cholesterol risk | | 0.99 (0.91-1.09) | | 2095/13636 | | 0.99 (0.90-1.08) | |
| High inflammatory risk | | 1.47 (1.34-1.62) | | 921/4004 | | 1.42 (1.28-1.57) | |
| Combined high risk | | 1.27 (1.16-1.38) | | 3141/13703 | | 1.17 (1.08-1.28) | |
| **Heart failure hospitalization** | |  | |  | |  | |
| Low risk | | ref. | | 413/5351 | | ref. | |
| High cholesterol risk | | 1.00 (0.89-1.12) | | 1059/13636 | | 0.99 (0.88-1.12) | |
| High inflammatory risk | | 1.38 (1.22-1.56) | | 551/4004 | | 1.30 (1.14-1.48) | |
| Combined high risk | | 1.21 (1.08-1.35) | | 1672/13703 | | 1.17 (1.04-1.31) | |
| **CV mortality** | |  | |  | |  | |
| Low risk | | ref. | | 155/5351 | | ref. | |
| High cholesterol risk | | 1.04 (0.87-1.25) | | 508/13636 | | 1.03 (0.86-1.24) | |
| High inflammatory risk | | 1.39 (1.14-1.70) | | 209/4004 | | 1.26 (1.02-1.55) | |
| Combined high risk | | 1.44 (1.21-1.71) | | 867/13703 | | 1.31 (1.10-1.57) | |

^a^ adjusted for age, sex, time since ASCVD, eGFR (as continuous variable), albuminuria, comorbidities (diabetes mellitus, hypertension, chronic respiratory disease, cancer, MI, angina, heart failure, peripheral vascular disease, stroke/TIA, atrial fibrillation, and rheumatoid diseases), undertaken procedures (coronary artery bypass grafting and percutaneous coronary intervention), and ongoing medications (antiplatelet, NSAIDs, angiotensin‐converting enzyme inhibitors/angiotensin receptor blockers, mineralocorticoid‐receptor antagonists, β blocker, SGLT-2i, diuretics, calcium channel blockers, digoxin, lipid-lowering treatment [statins, PCSK9i, ezetimibe]).

**Table S9**. Sensitivity analysis: number of events and hazard ratios for the risk of adverse outcomes associated with baseline hsCRP categories after exclusion of early events (within the first 6 or 12 months of follow-up).

|  |  | | | | **After excluding events within 6 months** | | | | | | | | **After excluding events within 12 months** | | | | |  |
| --- | --- | --- | --- | --- | --- | --- | --- | --- | --- | --- | --- | --- | --- | --- | --- | --- | --- | --- |
|  | **Original analysis**  **(for comparison)**  **Adj. HR (95% CI)^a^** | | | | **Number of events/**  **number of patients** | | | **Adj. HR (95% CI)^a^** | | | **Number of events/**  **number of patients** | | | | **Adj. HR (95% CI)^a^** | | |  |
| **MACE** | | |  |  | | |  | | |  | | | |  | | |  |  |
| Low risk | | ref. | | | | 414/5279 | | | ref. | | | 361/5226 | | | | ref. | | |
| High cholesterol risk | | 1.12 (1.01-1.24) | | | | 1411/13487 | | | 1.14 (1.02-1.27) | | | 1260/13336 | | | | 1.12 (0.99-1.26) | | |
| High inflammatory risk | | 1.18 (1.05-1.33) | | | | 542/4638 | | | 1.17 (1.03-1.33) | | | 466/4562 | | | | 1.17 (1.02-1.34) | | |
| Combined high risk | | 1.39 (1.26-1.54) | | | | 2285/15546 | | | 1.38 (1.24-1.54) | | | 2024/15285 | | | | 1.37 (1.22-1.54) | | |
| **All-cause mortality** | |  | | | |  | | |  | | |  | | | |  | | |
| Low risk | | ref. | | | | 604/5306 | | | ref. | | | 560/5262 | | | | ref. | | |
| High cholesterol risk | | 0.99 (0.91-1.09) | | | | 2004/13545 | | | 1.00 (0.92-1.10) | | | 1911/13452 | | | | 1.01 (0.92-1.12) | | |
| High inflammatory risk | | 1.47 (1.34-1.62) | | | | 1071/4594 | | | 1.43 (1.30-1.59) | | | 948/4471 | | | | 1.40 (1.26-1.55) | | |
| Combined high risk | | 1.27 (1.16-1.38) | | | | 3650/15567 | | | 1.25 (1.14-1.37) | | | 3368/15285 | | | | 1.23 (1.13-1.35) | | |
| **Heart failure hospitalization** | |  | | | |  | | |  | | |  | | | |  | | |
| Low risk | | ref. | | | | 329/5267 | | | ref. | | | 288/5226 | | | | ref. | | |
| High cholesterol risk | | 1.00 (0.89-1.12) | | | | 918/13495 | | | 1.01 (0.89-1.15) | | | 822/13399 | | | | 0.99 (0.86-1.13) | | |
| High inflammatory risk | | 1.38 (1.22-1.56) | | | | 545/4563 | | | 1.38 (1.20-1.58) | | | 444/4462 | | | | 1.32 (1.13-1.53) | | |
| Combined high risk | | 1.21 (1.08-1.35) | | | | 1726/15546 | | | 1.22 (1.08-1.38) | | | 1488/15308 | | | | 1.18 (1.04-1.35) | | |
| **CV mortality** | |  | | | |  | | |  | | |  | | | |  | | |
| Low risk | | ref. | | | | 604/5306 | | | ref. | | | 560/5262 | | | | ref. | | |
| High cholesterol risk | | 1.04 (0.87-1.25) | | | | 2004/13545 | | | 1.00 (0.92-1.10) | | | 1911/13452 | | | | 1.01 (0.92-1.12) | | |
| High inflammatory risk | | 1.39 (1.14-1.70) | | | | 1071/4594 | | | 1.43 (1.30-1.59) | | | 948/4471 | | | | 1.40 (1.26-1.55) | | |
| Combined high risk | | 1.44 (1.21-1.71) | | | | 3650/15567 | | | 1.25 (1.14-1.37) | | | 3368/15285 | | | | 1.23 (1.13-1.35) | | |

^a^ adjusted for age, sex, time since ASCVD, eGFR (as continuous variable), albuminuria, comorbidities (diabetes mellitus, hypertension, chronic respiratory disease, cancer, MI, angina, heart failure, peripheral vascular disease, stroke/TIA, atrial fibrillation, and rheumatoid diseases), undertaken procedures (coronary artery bypass grafting and percutaneous coronary intervention), and ongoing medications (antiplatelet, NSAIDs, angiotensin‐converting enzyme inhibitors/angiotensin receptor blockers, mineralocorticoid‐receptor antagonists, β blocker, SGLT-2i, diuretics, calcium channel blockers, digoxin, lipid-lowering treatment [statins, PCSK9i, ezetimibe]).

**Table S10.** Sensitivity analysis: number of events and hazard ratios for the risk of adverse outcomes after exclusion of patients with chronic inflammatory conditions (rheumatoid diseases or inflammatory bowel disease).

|  |  | | | | **After excluding patients with rheumatoid diseases or inflammatory bowel disease** | | | |  |  |
| --- | --- | --- | --- | --- | --- | --- | --- | --- | --- | --- |
|  | **Original analysis**  **(for comparison)**  **Adj. HR (95% CI)^a^** | | | | **Number of events/**  **number of patients** | | | **Adj. HR (95% CI)^a^** | | |
| **MACE** | |  |  | | |  |  |  |  |  |
| Low risk | ref. | | | 418/4,691 | | | ref. | | |  |
| High cholesterol risk | 1.12 (1.01-1.24) | | | 1,386/12,232 | | | 1.12 (1.00-1.25) | | |  |
| High inflammatory risk | 1.18 (1.05-1.33) | | | 540/3,843 | | | 1.19 (1.05-1.36) | | |  |
| Combined high risk | 1.39 (1.26-1.54) | | | 2,148/13,321 | | | 1.38 (1.24-1.54) | | |  |
| **All-cause mortality** |  | | |  | | |  | | |  |
| Low risk | ref. | | | 129/4,691 | | | ref. | | |  |
| High cholesterol risk | 0.99 (0.91-1.09) | | | 439/12,232 | | | 1.04 (0.85-1.27) | | |  |
| High inflammatory risk | 1.47 (1.34-1.62) | | | 245/3,843 | | | 1.44 (1.16-1.79) | | |  |
| Combined high risk | 1.27 (1.16-1.38) | | | 890/13,321 | | | 1.43 (1.18-1.73) | | |  |
| **Heart failure hospitalization** |  | | |  | | |  | | |  |
| Low risk | ref. | | | 340/4,691 | | | ref. | | |  |
| High cholesterol risk | 1.00 (0.89-1.12) | | | 916/12,232 | | | 1.03 (0.90-1.17) | | |  |
| High inflammatory risk | 1.38 (1.22-1.56) | | | 546/3,843 | | | 1.35 (1.17-1.54) | | |  |
| Combined high risk | 1.21 (1.08-1.35) | | | 1,631/13,321 | | | 1.26 (1.11-1.42) | | |  |
| **CV mortality** |  | | |  | | |  | | |  |
| Low risk | ref. | | | 129/4,691 | | | ref. | | |  |
| High cholesterol risk | 1.04 (0.87-1.25) | | | 439/12,232 | | | 1.04 (0.85-1.27) | | |  |
| High inflammatory risk | 1.39 (1.14-1.70) | | | 245/3,843 | | | 1.44 (1.16-1.79) | | |  |
| Combined high risk | 1.44 (1.21-1.71) | | | 890/13,321 | | | 1.43 (1.18-1.73) | | |  |

^a^ Adjusted for age, sex, time since ASCVD, eGFR (as continuous variable), albuminuria, comorbidities (diabetes mellitus, hypertension, chronic respiratory disease, cancer, MI, angina, heart failure, peripheral vascular disease, stroke/TIA, atrial fibrillation), undertaken procedures (coronary artery bypass grafting and percutaneous coronary intervention), and ongoing medications (antiplatelet, NSAIDs, angiotensin-converting enzyme inhibitors/angiotensin receptor blockers, mineralocorticoid-receptor antagonists, β blocker, SGLT-2i, diuretics, calcium channel blockers, digoxin, lipid-lowering treatment [statins, PCSK9i, ezetimibe]).

*Note: This analysis excludes 5,551 patients with rheumatoid diseases (n=5,030) or inflammatory bowel disease (n=636), with some overlap. Total patients in analysis: N=34,087.*

**Table S11**. Subgroup analysis: Association of high inflammatory and cholesterol risk categories with adverse cardiovascular outcomes, stratified by use of LLT

|  | **Off LLT**  (N=15,400) | | **On LLT**  (N=24,238) | | **P value** | |  |
| --- | --- | --- | --- | --- | --- | --- | --- |
|  | **No. of events/No. of patients** | **Adj. HR (95% CI)^a^** | **No. of events/No. of patients** | **Adj. HR (95% CI) ^a^** | |  | |
| **Major adverse cardiovascular events** |  |  |  |  |  | |  |
| Low risk | 378/4380 | ref. | 108/971 | ref. | 0.798 | |  |
| High cholesterol risk | 890/7975 | 1.14 (0.93-1.40) | 670/5661 | 1.11 (0.98-1.26) |  | |  |
| High inflammatory risk | 458/3616 | 1.24 (0.98-1.57) | 197/1135 | 1.16 (1.01-1.33) |  | |  |
| Combined high risk | 1323/8267 | 1.45 (1.18-1.77) | 1316/7633 | 1.38 (1.22-1.55) |  | |  |
| **All-cause mortality** |  |  |  |  |  | |  |
| Low risk | 477/4380 | ref. | 172/971 | ref. | 0.265 | |  |
| High cholesterol risk | 1089/7975 | 0.90 (0.76-1.06) | 1006/5661 | 1.03 (0.93-1.15) |  | |  |
| High inflammatory risk | 826/3616 | 1.41 (1.18-1.69) | 402/1135 | 1.49 (1.32-1.67) |  | |  |
| Combined high risk | 1819/8267 | 1.17 (1.00-1.37) | 2164/7633 | 1.30 (1.17-1.44) |  | |  |
| **Heart failure hospitalization** |  |  |  |  |  | |  |
| Low risk | 311/4380 | ref. | 102/971 | ref. | 0.564 | |  |
| High cholesterol risk | 583/7975 | 0.89 (0.72-1.11) | 476/5661 | 1.02 (0.89-1.18) |  | |  |
| High inflammatory risk | 526/3616 | 1.21 (0.95-1.54) | 207/1135 | 1.42 (1.23-1.64) |  | |  |
| Combined high risk | 1017/8267 | 1.09 (0.89-1.35) | 1063/7633 | 1.25 (1.10-1.43) |  | |  |
| **CV mortality** |  |  |  |  |  | |  |
| Low risk | 114/4380 | ref. | 41/971 | ref. | 0.454 | |  |
| High cholesterol risk | 271/7975 | 0.90 (0.64-1.26) | 237/5661 | 1.13 (0.91-1.41) |  | |  |
| High inflammatory risk | 200/3616 | 1.29 (0.89-1.87) | 100/1135 | 1.41 (1.12-1.79) |  | |  |
| Combined high risk | 485/8267 | 1.36 (0.99-1.88) | 640/7633 | 1.44 (1.17-1.77) |  | |  |

^a^ adjusted for age, sex, time since ASCVD, eGFR, albuminuria, comorbidities (diabetes mellitus, hypertension, chronic respiratory disease, cancer, MI, angina, heart failure, peripheral vascular disease, stroke/TIA, atrial fibrillation, and rheumatoid diseases), undertaken procedures (coronary artery bypass grafting and percutaneous coronary intervention), and ongoing medications (antiplatelet, NSAIDs, angiotensin‐converting enzyme inhibitors/angiotensin receptor blockers, mineralocorticoid‐receptor antagonists, β blocker, SGLT-2i, diuretics, calcium channel blockers, digoxin).

**Table S12**. Subgroup analysis: Association of high inflammatory and cholesterol risk categories with adverse cardiovascular outcomes, stratified by baseline eGFR categories

|  | **eGFR ≥60mL/min/1.73 m^2^** (N=31,781) | | **eGFR <60mL/min/1.73 m^2^** (N=7,857) | | **P value** |
| --- | --- | --- | --- | --- | --- |
|  | **No. of events/No. of patients** | **Adj. HR (95% CI)^a^** | **No. of events/No. of patients** | **Adj. HR (95% CI) ^a^** |  |
| **Major adverse cardiovascular events** |  |  |  |  | 0.112 |
| Low risk | 332/4421 | ref. | 154/930 | ref. |  |
| High cholesterol risk | 1156/11566 | 1.15 (1.01-1.30) | 404/2070 | 1.08 (0.89-1.31) |  |
| High inflammatory risk | 384/3435 | 1.21 (1.04-1.40) | 271/1316 | 1.08 (0.89-1.33) |  |
| Combined high risk | 1744/12359 | 1.45 (1.29-1.64) | 895/3541 | 1.27 (1.06-1.52) |  |
| **All-cause mortality** |  |  |  |  | <0.001 |
| Low risk | 410/4421 | ref. | 239/930 | ref. |  |
| High cholesterol risk | 1414/11566 | 0.99 (0.89-1.11) | 681/2070 | 1.04 (0.89-1.20) |  |
| High inflammatory risk | 687/3435 | 1.57 (1.39-1.78) | 541/1316 | 1.28 (1.10-1.50) |  |
| Combined high risk | 2410/12359 | 1.30 (1.17-1.45) | 1573/3541 | 1.22 (1.06-1.40) |  |
| **Heart failure hospitalization** |  |  |  |  | 0.776 |
| Low risk | 257/4421 | ref. | 156/930 | ref. |  |
| High cholesterol risk | 693/11566 | 0.96 (0.83-1.11) | 366/2070 | 1.04 (0.86-1.26) |  |
| High inflammatory risk | 381/3435 | 1.30 (1.11-1.53) | 352/1316 | 1.42 (1.17-1.72) |  |
| Combined high risk | 1198/12359 | 1.17 (1.02-1.35) | 882/3541 | 1.23 (1.03-1.47) |  |
| **CV mortality** |  |  |  |  | 0.318 |
| Low risk | 82/4421 | ref. | 73/930 | ref. |  |
| High cholesterol risk | 328/11566 | 1.14 (0.89-1.46) | 180/2070 | 0.93 (0.71-1.24) |  |
| High inflammatory risk | 147/3435 | 1.55 (1.18-2.03) | 153/1316 | 1.16 (0.87-1.54) |  |
| Combined high risk | 634/12359 | 1.58 (1.25-2.01) | 491/3541 | 1.25 (0.97-1.62) |  |

^a^ adjusted for age, sex, time since ASCVD, eGFR (as continuous variable), albuminuria, comorbidities (diabetes mellitus, hypertension, chronic respiratory disease, cancer, MI, angina, heart failure, peripheral vascular disease, stroke/TIA, atrial fibrillation, and rheumatoid diseases), undertaken procedures (coronary artery bypass grafting and percutaneous coronary intervention), and ongoing medications (antiplatelet, NSAIDs, angiotensin‐converting enzyme inhibitors/angiotensin receptor blockers, mineralocorticoid‐receptor antagonists, β blocker, SGLT-2i, diuretics, calcium channel blockers, digoxin, lipid-lowering treatment [statins, PCSK9i, ezetimibe]).

**Table S13**. Subgroup analysis by sex: number of events and hazard ratios for the risk of adverse outcomes associated with residual risk categories.

|  | **Men**  **(N=24,027)** | | **Women**  **(N=15,611)** | | **P value** |
| --- | --- | --- | --- | --- | --- |
|  | **No. of events/No. of patients** | **Adj. HR (95% CI)^a^** | **No. of events/No. of patients** | **Adj. HR (95% CI) ^a^** |  |
| **Major adverse cardiovascular events** |  |  |  |  | 0.364 |
| Low risk | 344/3,939 | ref. | 142/1,412 | ref. |  |
| High cholesterol risk | 940/8,035 | 1.14 (1.00-1.29) | 620/5,601 | 1.05 (0.87-1.26) |  |
| High inflammatory risk | 441/3,217 | 1.23 (1.07-1.42) | 214/1,534 | 1.09 (0.88-1.35) |  |
| Combined high risk | 1,528/8,836 | 1.47 (1.30-1.65) | 1,111/7,064 | 1.26 (1.06-1.51) |  |
|  |  |  |  |  |  |

^a^ adjusted for age, time since ASCVD, eGFR (as continuous variable), albuminuria, comorbidities (diabetes mellitus, hypertension, chronic respiratory disease, cancer, MI, angina, heart failure, peripheral vascular disease, stroke/TIA, atrial fibrillation, and rheumatoid diseases), undertaken procedures (coronary artery bypass grafting and percutaneous coronary intervention), and ongoing medications (antiplatelet, NSAIDs, angiotensin‐converting enzyme inhibitors/angiotensin receptor blockers, mineralocorticoid‐receptor antagonists, β blocker, SGLT-2i, diuretics, calcium channel blockers, digoxin, lipid-lowering treatment [statins, PCSK9i, ezetimibe]).

**Table S14**. Subgroup analysis by age: number of events and hazard ratios for the risk of adverse outcomes associated with residual risk categories.

|  | **Age <65 years**  (N=13,013) | | **Age ≥65 years**  (N=26,625) | | **P value** |
| --- | --- | --- | --- | --- | --- |
|  | **No. of events/No. of patients** | **Adj. HR (95% CI)^a^** | **No. of events/No. of patients** | **Adj. HR (95% CI) ^a^** |  |
| **Major adverse cardiovascular events** |  |  |  |  | 0.001 |
| Low risk | 96/1,856 | ref. | 390/3,495 | ref. |  |
| High cholesterol risk | 313/4,861 | 1.25 (0.99-1.58) | 1,247/8,775 | 1.10 (0.98-1.24) |  |
| High inflammatory risk | 114/1,341 | 1.41 (1.07-1.86) | 541/3,410 | 1.15 (1.00-1.31) |  |
| Combined high risk | 497/4,955 | 1.64 (1.31-2.06) | 2,142/10,945 | 1.34 (1.20-1.50) |  |
|  |  |  |  |  |  |

^a^ adjusted for age,sex, time since ASCVD, eGFR (as continuous variable), albuminuria, comorbidities (diabetes mellitus, hypertension, chronic respiratory disease, cancer, MI, angina, heart failure, peripheral vascular disease, stroke/TIA, atrial fibrillation, and rheumatoid diseases), undertaken procedures (coronary artery bypass grafting and percutaneous coronary intervention), and ongoing medications (antiplatelet, NSAIDs, angiotensin‐converting enzyme inhibitors/angiotensin receptor blockers, mineralocorticoid‐receptor antagonists, β blocker, SGLT-2i, diuretics, calcium channel blockers, digoxin, lipid-lowering treatment [statins, PCSK9i, ezetimibe]).

**Table S15**. Subgroup analysis: Association of high inflammatory and cholesterol risk categories with adverse cardiovascular outcomes, stratified by calendar year period

|  | **Pre 2016** (N=19,450) | | **2016-2021** (N=20,188) | | **P value** |
| --- | --- | --- | --- | --- | --- |
|  | **No. of events/No. of patients** | **Adj. HR (95% CI)^a^** | **No. of events/No. of patients** | **Adj. HR (95% CI) ^a^** |  |
| **Major adverse cardiovascular events** |  |  |  |  | <0.001 |
| Low risk | 302/1746 | ref. | 184/3605 | ref. |  |
| High cholesterol risk | 1221/7273 | 1.11 (0.97-1.26) | 339/6363 | 1.04 (0.87-1.26) |  |
| High inflammatory risk | 408/1795 | 1.12 (0.96-1.30) | 247/2956 | 1.28 (1.06-1.56) |  |
| Combined high risk | 2002/8636 | 1.36 (1.20-1.54) | 637/7264 | 1.39 (1.17-1.65) |  |
| **All Cause mortality** |  |  |  |  | <0.001 |
| Low risk | 434/1746 | ref. | 215/3605 | ref. |  |
| High cholesterol risk | 1733/7273 | 1.02 (0.92-1.14) | 362/6363 | 0.86 (0.72-1.00) |  |
| High inflammatory risk | 767/1795 | 1.35 (1.20-1.52) | 461/2956 | 1.72 (1.46-2.03) |  |
| Combined high risk | 3092/8636 | 1.24 (1.12-1.37) | 891/7264 | 1.33 (1.14-1.55) |  |
| **Heart failure hospitalization** |  |  |  |  | 0.001 |
| Low risk | 242/1746 | ref. |  | ref. |  |
| High cholesterol risk | 803/7273 | 1.01 (0.87-1.17) | 256/6363 | 0.90 (0.74-1.10) |  |
| High inflammatory risk | 417/1795 | 1.28 (1.09-1.51) | 316/2956 | 1.50 (1.24-1.81) |  |
| Combined high risk | 1515/8636 | 1.17 (1.02-1.35) | 565/7264 | 1.24 (1.03-1.48) |  |
| **CV mortality** |  |  |  |  | <0.001 |
| Low risk | 105/1746 | ref. | 434/7273 | ref. |  |
| High cholesterol risk | 434/7273 | 1.10 (0.89-1.37) | 199/1795 | 0.72 (0.50-1.04) |  |
| High inflammatory risk | 199/1795 | 1.34 (1.05-1.70) | 888/8636 | 1.44 (1.01-2.03) |  |
| Combined high risk | 888/8636 | 1.42 (1.16-1.75) | 434/7273 | 1.37 (1.00-1.89) |  |

^a^ adjusted for age, sex, time since ASCVD, eGFR (as continuous variable), albuminuria, comorbidities (diabetes mellitus, hypertension, chronic respiratory disease, cancer, MI, angina, heart failure, peripheral vascular disease, stroke/TIA, atrial fibrillation, and rheumatoid diseases), undertaken procedures (coronary artery bypass grafting and percutaneous coronary intervention), and ongoing medications (antiplatelet, NSAIDs, angiotensin‐converting enzyme inhibitors/angiotensin receptor blockers, mineralocorticoid‐receptor antagonists, β blocker, SGLT-2i, diuretics, calcium channel blockers, digoxin, lipid-lowering treatment [statins, PCSK9i, ezetimibe]).

## *Supplementary Figures*

**Figure S1**: Study design.


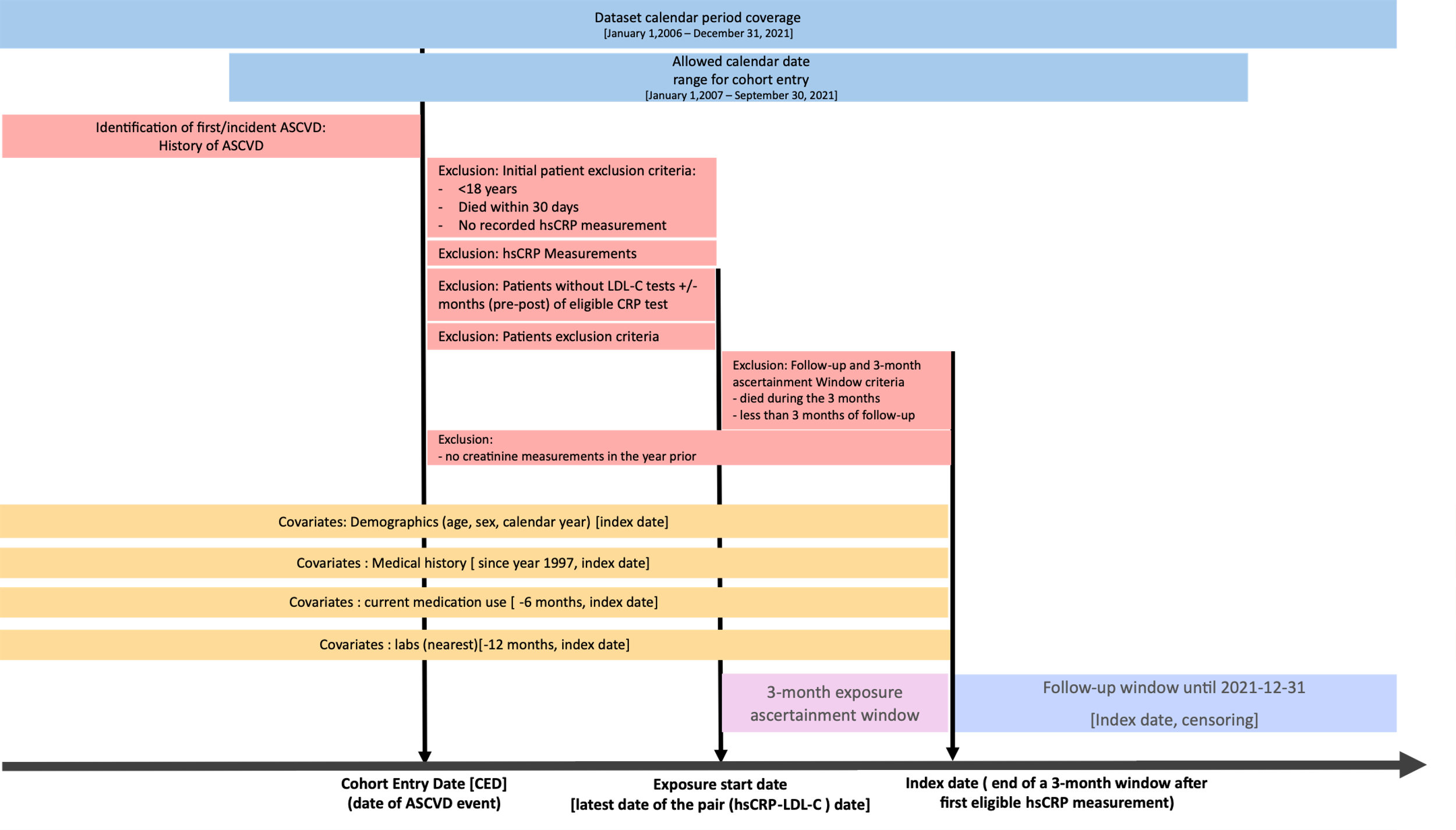


This graphical depiction of the study design shows the different time windows used to define exposure, exclusion criteria, baseline covariates, and outcomes.

See supplemental methods for detailed exclusion criteria.

Atherosclerotic cardiovascular disease; CED: cohort entry date; hsCRP: C-reactive protein; LDL-C: Low-density lipoprotein cholesterol.

**Figure S2:** Restricted cubic splines depicting the multivariable-adjusted hazard ratios (95% CIs) for major adverse cardiovascular events (MACE) according to baseline levels of (A) high sensitivity C-reactive protein (hsCRP) and (B) low-density lipoprotein cholesterol (LDL-C)


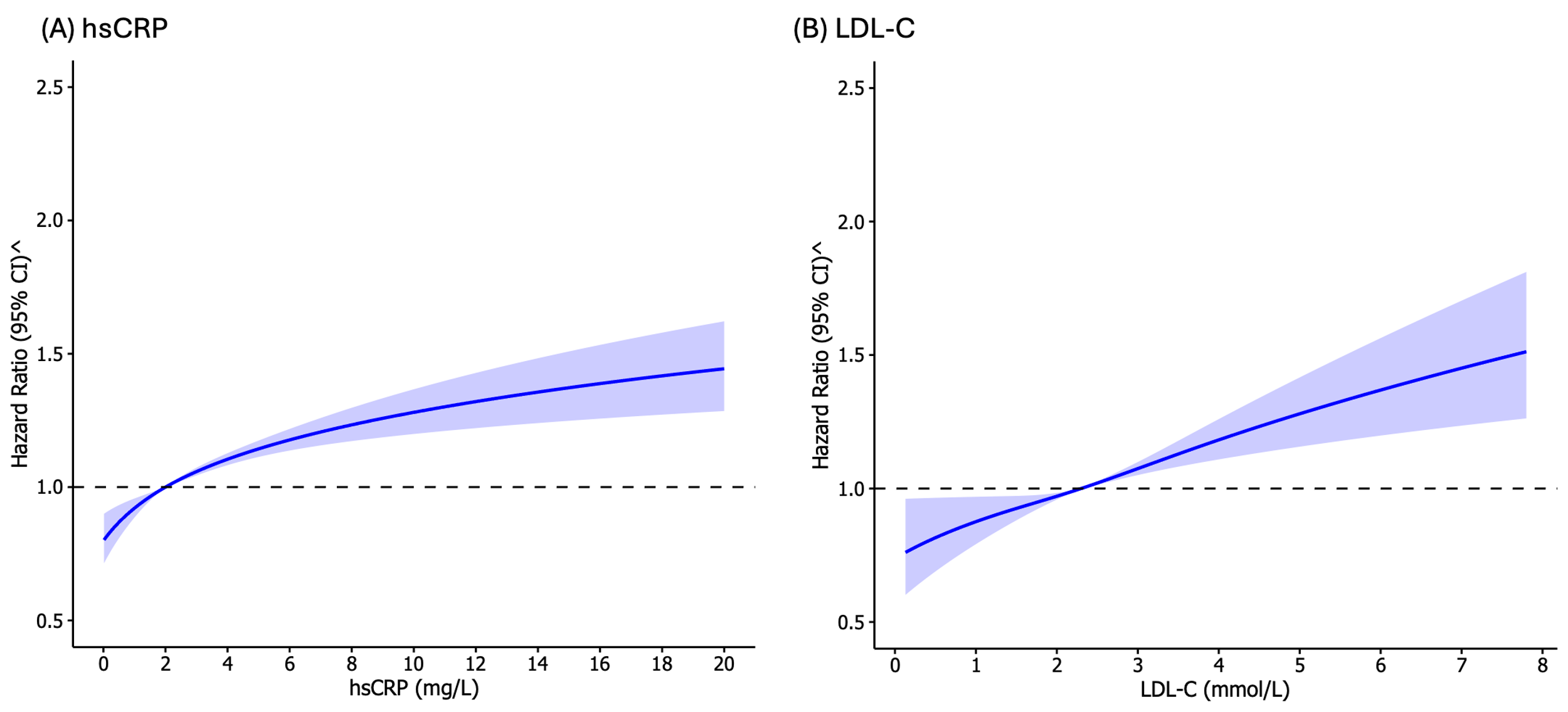


Solid lines represent hazard ratios; shaded areas represent 95% confidence intervals. Reference values were set at the median. P for non-linearity: hsCRP = 0.83; LDL-C = 0.34

^^^ adjusted for age, sex, time since ASCVD, eGFR (as continuous variable), albuminuria, comorbidities (diabetes mellitus, hypertension, chronic respiratory disease, cancer, MI, angina, heart failure, peripheral vascular disease, stroke/TIA, atrial fibrillation, and rheumatoid diseases), undertaken procedures (coronary artery bypass grafting and percutaneous coronary intervention), and ongoing medications (antiplatelet, NSAIDs, angiotensin‐converting enzyme inhibitors/angiotensin receptor blockers, mineralocorticoid‐receptor antagonists, β blocker, SGLT-2i, diuretics, calcium channel blockers, digoxin, lipid-lowering treatment [statins, PCSK9i, ezetimibe]).

**Figure S3:** Restricted cubic splines depicting the multivariable-adjusted hazard ratios (95% CIs) for the association between high sensitivity C-reactive protein (hsCRP) (A) and LDL cholesterol (LDL-C) (B) levels and risk of major adverse cardiovascular events (MACE), stratified by baseline lipid-lowering therapy (LLT) use


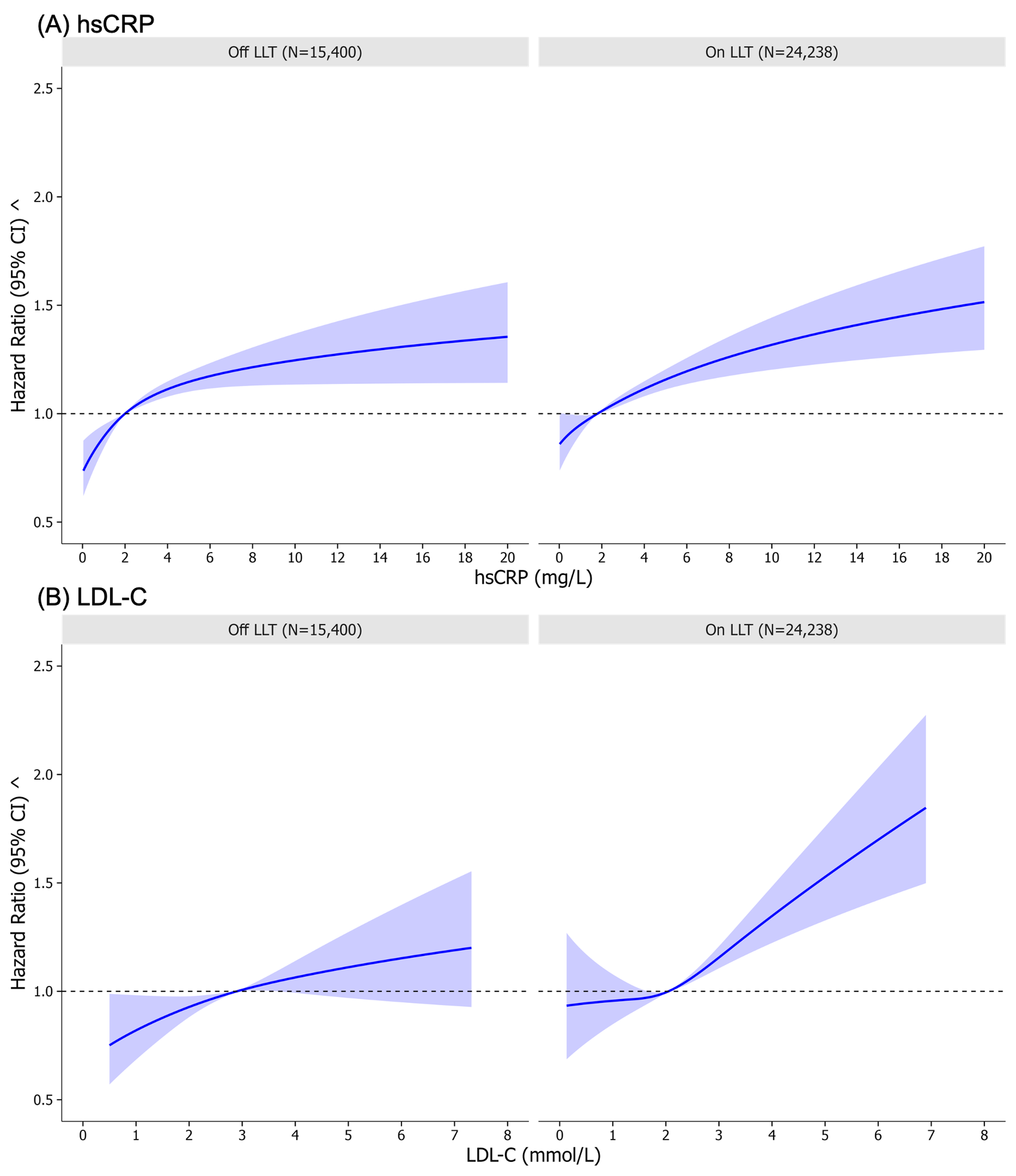


Solid lines represent hazard ratios; shaded areas represent 95% confidence intervals. Reference values were set at the median. P for non-linearity: hsCRP (On LLT) = 0.584, hsCRP (Off LLT) = 0.234; LDL-C (On LLT) = 0.012, LDL-C (Off LLT) = 0.831

^^^ adjusted for age, sex, time since ASCVD, eGFR (as continuous variable), albuminuria, comorbidities (diabetes mellitus, hypertension, chronic respiratory disease, cancer, MI, angina, heart failure, peripheral vascular disease, stroke/TIA, atrial fibrillation, and rheumatoid diseases), undertaken procedures (coronary artery bypass grafting and percutaneous coronary intervention), and ongoing medications (antiplatelet, NSAIDs, angiotensin‐converting enzyme inhibitors/angiotensin receptor blockers, mineralocorticoid‐receptor antagonists, β blocker, SGLT-2i, diuretics, calcium channel blockers, digoxin, lipid-lowering treatment [statins, PCSK9i, ezetimibe]).

**Figure S4:** Restricted cubic splines depicting the multivariable-adjusted HRs (95% CIs) for the association between high sensitivity C-reactive protein (hsCRP) and LDL-C levels and risk of major adverse cardiovascular events (MACE), stratified by eGFR categories


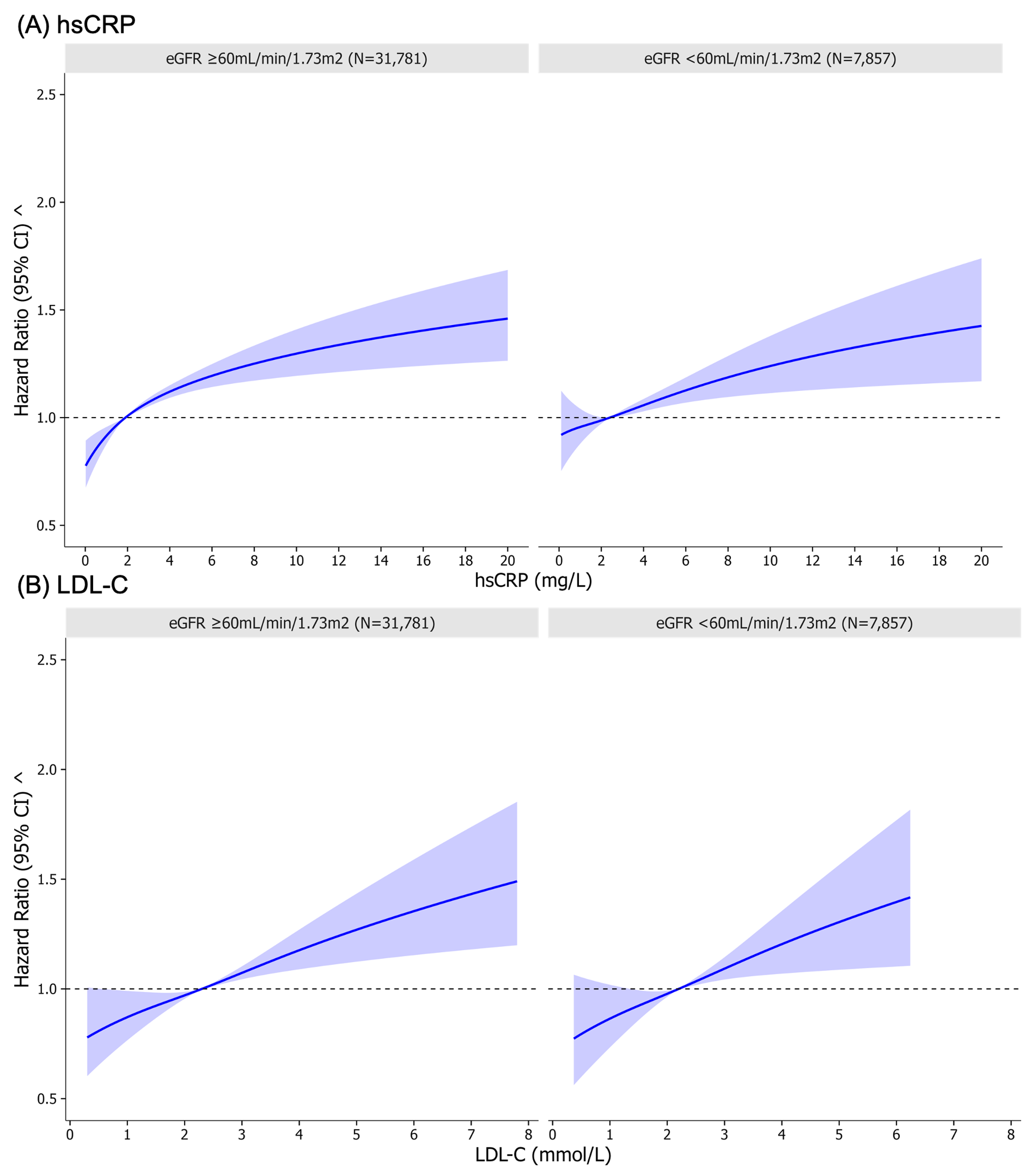


Solid lines represent hazard ratios; shaded areas represent 95% confidence intervals. Reference values were set at the median. P for non-linearity: hsCRP (eGFR ≥60) = 0.580, hsCRP (eGFR <60) = 0.346; LDL-C (eGFR ≥60) = 0.519, LDL-C (eGFR <60) = 0.671.

^^^ adjusted for age, sex, time since ASCVD, eGFR (as continuous variable), albuminuria, comorbidities (diabetes mellitus, hypertension, chronic respiratory disease, cancer, MI, angina, heart failure, peripheral vascular disease, stroke/TIA, atrial fibrillation, and rheumatoid diseases), undertaken procedures (coronary artery bypass grafting and percutaneous coronary intervention), and ongoing medications (antiplatelet, NSAIDs, angiotensin‐converting enzyme inhibitors/angiotensin receptor blockers, mineralocorticoid‐receptor antagonists, β blocker, SGLT-2i, diuretics, calcium channel blockers, digoxin).
